# A spontaneous mutation in *kdsD*, a biosynthesis gene for 3 Deoxy-_D_-*manno*-Octulosonic Acid, occurred in a ciprofloxacin resistant strain of *Francisella tularensis* and caused a high level of attenuation in murine models of tularemia

**DOI:** 10.1371/journal.pone.0174106

**Published:** 2017-03-22

**Authors:** Taylor Chance, Jennifer Chua, Ronald G. Toothman, Jason T. Ladner, Jonathan E. Nuss, Jo Lynne Raymond, Fabrice V. Biot, Samandra Demons, Lynda Miller, Stephanie Halasohoris, Sherry Mou, Galina Koroleva, Sean Lovett, Gustavo Palacios, Nicholas J. Vietri, Patricia L. Worsham, Christopher K. Cote, Todd M. Kijek, Joel A. Bozue

**Affiliations:** 1 Pathology Division, United States Army Medical Research Institute of Infectious Diseases (USAMRIID), Fort Detrick, Frederick, MD, United States of America; 2 Bacteriology Division, USAMRIID, Fort Detrick, Frederick, MD, United States of America; 3 Center for Genome Sciences, USAMRIID, Fort Detrick, Frederick, MD, United States of America; 4 Department of Molecular and Translational Sciences, USAMRIID, Fort Detrick, Frederick, MD, United States of America; 5 Institut de Recherche Biomédicale des Armées, Département de Biologie des Agents Transmissibles, Unité de Bactériologie/UMR_MD1, B.P. 73, Brétigny-sur-Orge, France; New York Medical College, UNITED STATES

## Abstract

*Francisella tularensis*, a gram–negative facultative intracellular bacterial pathogen, is the causative agent of tularemia and able to infect many mammalian species, including humans. Because of its ability to cause a lethal infection, low infectious dose, and aerosolizable nature, *F*. *tularensis* subspecies *tularensis* is considered a potential biowarfare agent. Due to its *in vitro* efficacy, ciprofloxacin is one of the antibiotics recommended for post-exposure prophylaxis of tularemia. In order to identify therapeutics that will be efficacious against infections caused by drug resistant select-agents and to better understand the threat, we sought to characterize an existing ciprofloxacin resistant (CipR) mutant in the Schu S4 strain of *F*. *tularensis* by determining its phenotypic characteristics and sequencing the chromosome to identify additional genetic alterations that may have occurred during the selection process. In addition to the previously described genetic alterations, the sequence of the CipR mutant strain revealed several additional mutations. Of particular interest was a frameshift mutation within *kdsD* which encodes for an enzyme necessary for the production of 3-Deoxy-_D_-*manno*-Octulosonic Acid (KDO), an integral component of the lipopolysaccharide (LPS). A *kdsD* mutant was constructed in the Schu S4 strain. Although it was not resistant to ciprofloxacin, the *kdsD* mutant shared many phenotypic characteristics with the CipR mutant, including growth defects under different conditions, sensitivity to hydrophobic agents, altered LPS profiles, and attenuation in multiple models of murine tularemia. This study demonstrates that the KdsD enzyme is essential for *Francisella* virulence and may be an attractive therapeutic target for developing novel medical countermeasures.

## Introduction

*Francisella tularensis* is a gram-negative bacterium that causes the life threatening and debilitating disease tularemia. As a facultative intracellular pathogen, its ability to replicate within various host cells, such as macrophages, dendritic cells, neutrophils, and epithelial cells is well documented and essential for virulence [1–13]. *F*. *tularensis* is able to infect a wide range of animal species, including humans. *F*. *tularensis* can be transmitted to humans through a number of routes; the most common being the bite of an infected insect or other arthropod vector [14–17]. Human illness can range from the ulceroglandular form to more serious pneumonic or typhoidal tularemia [15]. In pneumonic tularemia, infection progresses from the lungs to other organs, primarily the liver and spleen [18–23]. The risk of infection is associated mainly with two subspecies, the more virulent *F*. *tularensis* ssp. *tularensis* (type A) and the less virulent *F*. *tularensis* ssp. *holarctica* (type B).

Due to its high pathogenicity, low infectious dose, and aerosizable nature, *F*. *tularensis* poses a serious potential threat for use as a biological weapon and therefore is classified by the US Department of Health and Human Services as a Tier 1 Select Agent [18, 20, 24, 25]. This threat is of even greater concern with the potential for development of antibiotic resistant strains of *Francisella* which has previously been demonstrated [26–28].

One of the major virulence factors of *Francisella* is lipopolysaccharide (LPS) which plays an important role in evasion of the host immune responses [29–33]. LPS is the major outer surface structure of gram-negative bacteria and consists of three components: lipid A, a polysaccharide core, and the O-antigen polysaccharide [34]. The core region of the LPS is linked to lipid A by 3-Deoxy-_D_-*manno*-Octulosonic Acid (KDO), an eight carbon sugar. The LPS of *F*. *tularensis* does not bind to the LPS binding protein or activate the Toll-like-receptor (TLR) 4 signaling pathway [35, 36]. In contrast, lipid A moieties from other gram-negative bacteria are able to interact with the TLR4, activating the innate immune system to stimulate a strong proinflammatory response [30, 36–38]. The inertness of *F*. *tularensis* LPS is speculated to be due to the atypical lipid A structure that is distinct from other gram-negative bacteria. Specifically, *F*. *tularensis* lipid A is asymmetrical and tetraacylated, possesses longer length fatty acid chains, lacks phosphate substituents, and contains a unique amino sugar moiety [29, 31, 34, 39–42].

The traditional therapy for tularemia is streptomycin, tetracycline, or doxycycline [19, 43–46]. However, the fluorinated quinolone, ciprofloxacin, may offer advantages as a first-line therapy of treatment of tularemia and is recommended as an acceptable treatment option for *F*. *tularensis*, particularly after an aerosol exposure resulting from the use as a biological weapon [18, 47–53]. The advantages for the use of ciprofloxacin over other antibiotics are the bactericidal effects, the potential for oral administration, and demonstrated *in vitro* activity [45, 54, 55]. Ciprofloxacin targets the bacterial type II enzymes, DNA gyrase (GyrA and GyrB) and topoisomerase IV (ParC and ParE) [56–59] and functions by stabilizing an intermediate stage of the DNA replication reaction thus inhibiting cell division [58, 60, 61]. Resistance to ciprofloxacin is caused by changes to the amino acid sequences around the enzyme active site resulting in reduced drug affinity and continued gyrase/topoisomerase activity thereby allowing for continued bacterial cell growth [58, 59].

In a previous study, a *F*. *tularensis* ciprofloxacin resistant (CipR) mutant of Schu S4 was generated by serially passaging on increasing concentrations of the antibiotic [26]. The CipR mutant contained two non-synonymous substitutions in *gyrA* and a five base pair (bp) deletion in *parE*. In the current study, we further characterized the phenotype of the Schu S4 CipR mutant and, more importantly, determined if this strain retained virulence. The genome was sequenced to identify other genetic alterations which occurred during the selection process, excluding those previously described to *gyrA* and *parE*. Interestingly, one of the other mutations to the CipR mutant strain was a frameshift in the *kdsD* gene which encodes for _D_- arabinose 5-phosphate isomerase. KdsD is an enzyme that catalyzes the conversion of the pentose pathway intermediate _D_-ribulose 5-phosphate (R5P) into D-arabinose 5-phosphate (A5P) [62]. A5P is a precursor of KDO, an integral part of the LPS, in which the lipid A-KDO molecule serves as a linker for the O-antigen polysaccharide [38]. As LPS is known to be an important virulence factor for *F*. *tularensis* [63–68], we sought to determine if the mutation of the *kdsD* gene led to many of the characteristics observed for the CipR mutant strain, such as the lack of an O-antigen and loss of virulence in various murine models of tularemia. We found that many of the phenotypes observed with the *kdsD* mutant were similar to those of CipR mutant.

## Materials and methods

### Bacterial strains

All strains and plasmids used in this study are listed in Table 1. *Escherichia coli* NEB Turbo cells (New England Biolabs) were used for cloning purposes. *E*. *coli* was propagated in Luria broth or agar supplemented with ampicillin at 100 μg/ml, hygromycin at 200 μg/ml, or kanamycin at 20 μg/ml as necessary. All cultures were grown at 37°C.

**Table 1:** Bacterial strains and plasmids.

	Relevant Characteristics	Reference/ Source
***E*. *coli***		
NEB Turbo	Cloning strain	NEB
***F*. *tularensis***		
Schu S4	Fully virulent Type A strain	USAMRIID collection
CipR (Ft-127)	2 bp substitutions in *gyrA* and a 5 bp deletion in *parE*; ciprofloxacin resistant	[26]
*kdsD*::*ltrB*_*L1*_	Inactivated *kdsD*	This study
*kdsD*::*ltrB*_*L1*_ with pMP831+*kdsD*	Complemented *kdsD* mutant strain	This study
***F*. *novicida***		
U112 strain	*F*. *tularensis* subsp. *novicida*	ATCC 15482 [15]
*kpsF*::T20	Inactivated *kpsF* (BEI catalog # NR-6746)	BEI [69]
*kpsF*::T20 with pMP831+*kdsD*	Complemented *kpsF*::T20	This study
**MIC analysis strains**		
*E*. *coli* ATCC 25922	Used as a standard for MIC quality control	ATCC
*S*. *aureus* ATCC 29213	Used as a standard for MIC quality control	ATCC
*P*. *aeruginosa* ATCC 27853	Used as a standard for MIC quality control	ATCC
**Plasmids**		
pKEK1140	Targetron plasmid	[70]
pKEK1140-*kdsD*	pKEK1140-tgt *kdsD* gene	This study
pMP831	Complementation plasmid	[71]
pMP831+*kdsD*	Plasmid containing the intact *Ft kdsD* gene	This study

MIC, minimum inhibitory concentration

The *F*. *tularensis* subsp.*tularensis* strains used included the fully virulent Schu S4 [23] and the CipR mutant Schu S4 derivative [26] which had been previously selected with approval by the Centers for Disease Control. Previous characterization of the CipR mutant strain determined that the *gyrA* gene contained two base pair (bp) substitutions: C248→T and G259→T. In addition, a five-bp deletion occurred in the *parE* gene. Also included in the current study was *F*. *tularensis* subsp. *novicida* strain U112 and a transposon derivate [69] (BEI).

For routine growth of *F*. *tularensis* species, bacteria were grown on enriched chocolate agar plates obtained from Remel^TM^ (product number R01300; Lenexa, KS). When necessary, agar was supplemented with kanamycin at 10 μg/ml and/ or hygromycin at 200 μg/ml. As indicated, *F*. *tularensis* was grown in broth culture in Chamberlains Defined Medium (CDM) [72] or brain heart infusion (BHI) broth supplemented with 1% Isovitalex (Becton Dickinson, Cockeysville, MD, USA).

USAMRIID is compliant with all federal and Department of Defense regulations pertaining to the use of Select Agents.

### Genomic sequencing and analysis

Genomic DNA was prepared from the ciprofloxacin resistant *F*. *tularensis* using the Qiagen Genomic-tip 500/G kit with the appropriate buffers according to the manufacturer’s instructions. DNA was sequenced on a Pacific Biosciences RSII. Specifically, the sequencing library was prepared using the SMRTbell™ Template Prep Kit (Pacific Biosciences, Menlo Park, CA) following manufacturer’s protocol. DNA (5 μg) was fragmented using gTUBE (Covaris Inc., Woburn, MA) to ~20 kb. After DNA damage repair and ends repair, blunt hairpin adapters were ligated to the template, and failed ligation products were digested with ExoIII and ExoVII exonucleases. Resulting SMRTbell template was size selected on BluePippin system (Sage Science, Beverly, MA) using 0.75% dye-free agarose cassette with 4-10kb Hi-Pass protocol and low cut set on 4 kb. Size selected template was cleaned and concentrated with AMPure PB beads. The P4 polymerase was used in combination with the C2 sequencing kit and we collected 240-minute movies. Raw reads were quality filtered (subread length > = 500; polymerase read quality > = 0.80) and assembled using HGAP 2 v2.1.0 with a length cutoff of 14,211 bp [73]. Gepard v1.30 [74] was used to identify repetitive, low-quality sequence at the contig ends, which was trimmed using custom scripts. The final genome assembly (Genbank: CP013853) was annotated using NCBI’s Prokaryotic Genome Annotation Pipeline v3.0 [75].

To identify genomic differences in *F*. *tularensis* CipR mutant relative to its parent strain, wgsim (github.com/lh3/wgsim) was used to computationally “shred” the *de novo* assembly into 1 million perfect-match read pairs (150bp x 2 with a fragment size of 500bp), for an average of ~150x depth. These synthetic reads were then aligned to the *F*. *tularensis* Schu S4 reference genome (Genbank: NC_006570) using Bowtie2 (reads were ignored if they mapped equally well to multiple places in the reference genome) [76] and variants were called using the UnifiedGenotyper in GATK v3.1-1-g07a4bf8 [77]. The predicted effects of variants were annotated with SnpEff [78] using the " Francisella_tularensis_SCHU_S4_uid57589" database.

### Mutant construction

The *kdsD*::*ltrB*_*L1*_ mutant strain of *F*. *tularensis* were created using a modified TargeTron (Sigma-Aldrich, St. Louis, MO) mutagenesis system [70]. In brief, the coding sequence of the gene of interest was entered into the Sigma TargeTron primer design site to determine the appropriate oligonucleotides for retargeting the intron. The modification to this procedure was an *Xho*I restriction site was substituted for the *Hind*III. The resulting PCR product was cloned into vector pKEK1140 [70]. The plasmid was introduced into the Schu S4 strain by electroporation and the transformed strains with the retargeted plasmid were grown at 30°C on chocolate agar with 10 μg/ml kanamycin. Kanamycin resistant colonies were then isolated and screened via PCR to identify mutant strains (Table 2). The presence of the TargeTron insertion was determined using an intron-specific EBS universal primer combined with a gene specific primer, and intron insertion of the targeted gene was determined using gene-specific primers that amplified across the insertion site (Table 2). To cure the plasmid from the mutant clones, bacteria were grown overnight at 39^°^C in BHI containing 1% Isovitalex and serially diluted on chocolate agar plates. Individual colonies were screened for loss of the pKEK1140 by PCR analysis (Table 2) and sensitivity to kanamycin (present on pKEK1140).

**Table 2:** Oligonucleotides used in this study.

Oligonucleotide	Sequence
611|612s-IBS	AAAA**CTCGAG**ATAATTATCCTTAGCATGCCCGCTAGTGCGCCCAGATAGGGTG
611|612s-EBS1d	CAGATTGTACAAATGTGGTGATAACAGATAAGTCCCGCTAAATAACTTACCTTTCTTTGT
611|612s-EBS2	TGAACGCAAGTTTCTAATTTCGATTCATGCTCGATAGAGGAAAGTGTCT
kdsD 5' cloning	*CGGACCG*GATTAATTTGAATATGTTTCAT
kdsD 3' cloning	*CGGACCG*GTTAGGTGATCCTGTAATGCTTA
Kan probe F	TGCATGGTTACTCACCACTGC
Kan probe R	TACAACCTATTAATTTCCCCTCG

Bolded sequence corresponds to *Xho*I restriction enzyme site. Underlined sequence corresponds *to BsrG*I restriction site. Italics sequence corresponds to *Rsr*II restriction enzyme site.

### Complementation of the *kdsD* mutation

For complementing the observed phenotypes from the *kdsD*::*ltrB*_*L1*_ *F*. *tularensis* and *kpsF*::T20 *F*. *novicida* mutant strains, a functional *kdsD* gene was PCR amplified from DNA from the Schu S4 strain with flanking upstream DNA which would presumably contains the promoter. The DNA fragment was cloned into vector pMP831 [71] and then transformed into the respective mutant strains by electroporation. The constructs were selected by hygromycin resistance (200 µg/ml) which is present on the vector.

#### Growth assays

Growth assays were performed in Chamberlains defined broth [72], with or without the addition of A5P (Sigma-Aldrich, product # A2013), as indicated. Assays were performed using an Infinite M200 Pro (Tecan; Männedorf, Switzerland) microplate reader in 96-well microtiter plates at 37^°^C with shaking. The OD_600_ was measured every 60 min. For all assays, *F*. *tularensis* or *F*. *novicida* strains were grown for 24 h or 18 h chocolate agar plate, respectively, and then resuspended in broth medium to an equal OD_600_. All samples were performed in quadruplicate and included medium controls to confirm sterility and for use as blanks to calculate the absorbance of the cultures.

### Macrophage assays

J774A.1 cells, a murine macrophage-like cell line obtained from the American Type Culture Collection, were seeded (~2.5x10^5^ cells/well) into 24-well plates and cultured 2 days (37°C, 5% CO_2_) at which time the cells had formed confluent monolayers. The cells were maintained in Dulbecco’s Modified Eagle’s medium (D-MEM) containing high glucose, 10% heat-inactivated fetal bovine serum (FBS), plus 1.5 g/l sodium bicarbonate. For the intracellular assays, *F*. *tularensis* or *F*. *novicida* was suspended in phosphate buffered saline (PBS) from a 24 h or 18 h plate, respectively, and then diluted 1:5 in tissue culture medium. The bacterial suspension was added to the macrophages in 200 μl to achieve a multiplicity of infection (MOI) of ~100:1, and the MOI was confirmed from this suspension by serial dilutions and plating on chocolate agar plates. The bacteria and macrophages were allowed to coincubate for 2 h at 37^°^C with 5% CO_2_. Next, the medium containing the extracellular bacteria was aspirated and replaced with fresh tissue culture medium supplemented with 25 μg/ ml of gentamycin for an additional 2 h. After this incubation, samples from the tissue culture wells were washed three times with PBS. The monolayer was then lysed with 200 μl of sterile water, immediately scraped, and suspended in 800 μl of PBS. The suspension was serially diluted in PBS and plated onto chocolate agar plates. The remaining tissue culture wells were assayed for CFU recovery at the 24 h post-challenge time point as described above. Replicate data from three separate experiments were normalized for comparing strains by determining the difference in percent CFU recovery between the assayed 4h and 24 h time points.

To analyze the fate of the macrophages infected with Schu S4 strains, coverslips containing the J774A.1 cells were fixed with 4% formalin, permeabilized with PBS containing 0.025% saponin and then subjected to Wright Giemsa solution (Electron Microscopy Sciences, Hatfield, PA) for 10 min. Coverslips were washed 3x with PBS and mounted. Light microscopy was performed on the Zeiss Axio Observer Z1 equipped with an x 40 oil objective lens, AxioCam HRc camera and Zen-Blue edition 2011 software (Carl Zeiss Microimaging, Thornwood, NY).

For analysis of macrophages infected with *F*. *novicida*, coverslips were removed and placed in media containing 1 drop of Cell Event Caspase 3/7 green ready probes reagent (ThermoFisher Scientific; Waltham, MA) and incubated for 30 min. Confocal microscopy was performed on the Zeiss 700 Laser Scanning Microscopy System using Zen-Black Edition 2011 software (Carl Zeiss Microimaging, Thornwood, NY). Fluorescent and differential interference contrast (DIC) images were collected using the ×40 (numerical aperture: 1.3) oil objective lens with the pinhole set to 2 Airy unit.

### Analysis of bacterial cell extracts

Whole-cell extracts were collected for protein and LPS analysis from plate grown *F*. *tularensis* and *F*. *novicida* strains. Cultures were prepared at equal colony forming unit (CFU) concentrations in PBS, lysed in gel loading buffer solution, and boiled for 30 min. Sterility of the extracts was confirmed. Proteins were fractioned on NuPage Novex 4–12% Bis-Tris gels. For western analysis, fractionated proteins were transferred onto a nitrocellulouse membrane using an iBlot Gel Transfer Device. After transfer, the membranes were blocked with 1% skim milk in Tris Buffered Saline + Tween 20. *F*. *tularensis* samples were blotted with mouse monoclonal antibodies, anti-LPS (F6070-02X; US Biological; Salem, MA) or anti-capsule (11B7; [63]), at a dilution of 1:500. *F*. *novicida* samples were blotted with a mouse monoclonal antibody from cell culture supernatants with an anti-LPS antibody, Fn#13, (ImmunoPrecise Antibodies; Victoria BC, Canada) at a dilution of 1:100. The loading control antibody used for all analyses was rabbit polyclonal anti-*E*.*coli* GroEL (dilution of 1:2,000) (Enzo Life Sciences; Farmingdale, NY). Bands were visualized using 3,3',5,5'-Tetramethylbenzidine Membrane Peroxidase substrate (Kirkegaard & Perry Laboratories, Inc; Gaithersburg, MD).

### Mass spectrometry analysis of lipid A

LPS from *F*. *tularensis* strains was prepared using a LPS extraction kit (Catalog # 17141) from Intron Biotechnology. Sterility of the LPS preparations was confirmed. The samples were analyzed by matrix-assisted laser desportion ionization time-of-flight (MALDI-TOF) mass spectrometry analysis using protocols developed by Zhou et al [79]. In short, 20 μl of each LPS sample was mixed with 80 μl of methanol/chloroform in a glass vial, briefly vortexed and 1 μl of the solubilized sample spotted on a stainless steel target. Samples were allowed to air dry and 0.5 μl of matrix (10 mg/ml 2,5-dihydrobenzoic acid) was added to each spot. Samples were analyzed by MALDI-TOF mass spectrometry in reflector/negative ion mode using an Applied Biosystems 5800 instrument (Foster City, CA). The instrument was calibrated with low molecular weight standards (Bruker; Billerica, MA) and data were collected from 800 to 4000 (m/z) by manual “hot spot” searching and adjusting laser intensity to obtain optimum signal to noise for each sample. Each of the reported spectra is averages of 1000 laser shots.

### Minimum inhibitory concentration (MIC) susceptibility assays

Ciprofloxacin was purchased from U.S. Pharmacopeia (Rockville, MD), made into 5 mg/mL stocks according to the CLSI guidelines (Clinical and Laboratory Standards Institute, 2013), and stored at -70^°^C until use. Bacterial inoculums were prepared by suspending colonies into cation-adjusted Mueller-Hinton broth (CAMHB) from isolates grown aerobically at 35^°^C on chocolate agar plates for 42–48 h. An inoculum was prepared to the density of a 0.5 McFarland and then diluted 1:100 with CAMHB to a bacterial cell density of ~10^6^ CFU/ml. To each well of the 96-well plate, 50 μl of the adjusted dilution was added for a final inoculum of ~5 x 10^4^ CFU/well. MICs were determined by the broth micro-dilution method in 96-well plates according to CLSI guidelines (M07-A10, June 2015). Ciprofloxacin was serially diluted two-fold in 50 μl of CAMHB. The antibiotic range tested was 0.03–64 μg/ml based on a final well volume of 100 μl after inoculation. Plates were incubated at 35^°^C and MICs determined visually at 42–48 h. Quality control was established by using *E*. *coli* ATCC 25922, *Staphylococcus aureus* ATCC 29213, and *Pseudomonas aeruginosa* ATCC 27853 according to CLSI guidelines.

### *In vitro* susceptibility assays

*F*. *tularensis* and *F*. *novicida* strains were suspended in PBS at an OD_600_ of ~ 0.2 and 100 μl aliquots were spread on chocolate agar plates. Sterile paper disks 10 mm in diameter were saturated in water, SDS (100 mg/ ml), Triton X-100 (5%), Tween 20 (5%), or polymyxin B (PMB) (10 mg/ ml), allowed to dry, and placed onto chocolate agar plates. For each study, three separate disks were prepared for each inhibitor and assessed by measuring the diameter of the zone of growth inhibition. The study was repeated three separate times.

### Animal challenges

To determine the ability to cause infection, BALB/c mice (8–9 week-old and obtained from Charles River Laboratories; Frederick, MD) were challenged with *F*. *tularensis* or *F*. *novicida* in groups of 10 by various routes. For all methods of infection, the challenge doses were determined by serial dilutions in PBS and plating on chocolate agar. Intradermal challenge. Frozen *F*. *tularensis* stocks were streaked onto chocolate agar and incubated at 37^°^C for 2 days. Next, a fresh chocolate agar plate was swabbed from the streak plate and grown for 24 h. Bacterial cells were harvested from the plate in PBS, and mice were challenged with 0.1 ml aliquots at various cell concentrations. Intranasal challenge. Mice were anesthetized with 150 μl of ketamine, acepromazine, and xylazine injected intramuscularly. The mice were then challenged by intranasal instillation with 50 μl of *F*. *tularensis* or *F*. *novicda* suspended in PBS from 24 h or 18 h, respectively, grown freshly from swabbed plate cultures. Aerosol challenge. For aerosol challenges, a 24 h swabbed plate was used to inoculate flasks containing 25 ml of BHI broth containing 1% Isovitalex at an approximate OD_600_ of 0.025. This medium was chosen for aerosol studies as it was previously shown to be more conducive for *Francisella* survival during aerosolization and improved spray factors [80]. The broth cultures were grown overnight at 37^°^C shaker at 150 rpm and adjusted for various challenge doses. Mice were exposed to *F*. *tularernsis* using a dynamic 30-liter humidity-controlled Plexiglas whole-body exposure chamber and calculated inhaled doses were obtained as previously described [81]. For all challenge experiments, mice were monitored several times each day and mortality rates (or euthanasia when moribund) were recorded. *In vivo* dissemination. For a *F*. *tularensis* dissemination study, mice were challenged intranasally as described above with the indicated strains and doses. At specified time points after challenge, mice were then euthanized within a CO_2_ chamber. The lungs and spleens were harvested, rinsed with PBS, weighed, and then homogenized in 1 ml of PBS in a disposable tissue grinder (Covidien; Mansfield, MA). The homogenates were then serially diluted and plated on to chocolate agar plates. For pathological analysis of the challenged mice over the course of the infection, additional mice (n = 3) were processed for histopathology as described below.

### Ethics statement

Challenged mice were observed at least twice daily for 21 days for clinical signs of illness. Humane endpoints were used during all studies, and mice were humanely euthanized when moribund according to an endpoint score sheet. Animals were scored on a scale of 0–12: 0–3 = no clinical signs; 4–7 = clinical signs; increase monitoring; 8–12 = distress; euthanize. Those animals receiving a score of 8–12 were humanely euthanized by CO_2_ exposure using compressed CO_2_ gas followed by cervical dislocation. However, even with multiple checks per day, some animals died as a direct result of the infection.

Animal research at The United States Army of Medical Research Institute of Infectious Diseases (USAMRIID) was conducted and approved under an Institutional Animal Care and Use Committee (USAMRIID IACUC) in compliance with the Animal Welfare Act, Public Health Service Policy, and other Federal statutes and regulations relating to animals and experiments involving animals. The facility where this research was conducted is accredited by the Association for Assessment and Accreditation of Laboratory Animal Care, International and adheres to principles stated in the Guide for the Care and Use of Laboratory Animals, National Research Council, 2011.

### Pathology

Postmortem tissues were collected from mice challenged with *F*. *tularensis*, fixed in 10% neutral buffered formalin, routinely processed, embedded in paraffin, and sectioned for hematoxylin and eosin (HE) staining. Tissues examined histopathologically included: nasal cavity, oropharyngeal cavity, salivary gland, brain, pituitary gland, eyes, external/middle/internal ear, submandibular lymph node, esophagus, trachea, lungs, heart, mediastinal lymph node, thyroid gland, thymus, liver, spleen, stomach, small intestine, large intestine, pancreas, mesenteric lymph node, adrenal gland, urinary bladder, uterus, ovary, and bone marrow. At least a single section of the above tissues were examined by a board certified veterinary pathologist and were subjectively graded on the severity of necrosis/inflammation: minimal (involving < 5% of the tissue), mild (involving 5–10% of the tissue), moderate (involving 11–25% of the tissue), marked (involving 26–50% of the tissue), or severe (involving > 50% of the tissue).

### Statistics

For comparing data from the sensitivity to inhibitor and CFU recovery from macrophages, statistical significance (*p*< 0.05) was determined by the two-tailed Student *t* test. Growth analysis of bacterial strains in broth media was analyzed as previously described [82]. We used a logistic growth equation to fit the data as a function of maximum density, lag time, and maximum growth rate. LD_50_ analysis was determined by the Bayesian probit analysis. Survival rates were compared between groups by Fisher exact tests with permutation adjustment for multiple comparisons using SAS Version 8.2 (SAS Institute Inc., SAS OnlineDoc, Version 8, Cary, N.C. 2000).

## Results

### The genome of the CipR mutant was sequenced and additional mutations were identified.

The CipR mutant was previously examined for mutations to genes that comprise the quinolone resistance-determining region, within which mutations frequently give rise to ciprofloxacin resistance [58, 59]. From the study by Loveless et al. [26], the CipR mutant was shown to contain two non-synonymous substitutions in *gyrA* and a five bp deletion in *parE*. To determine if other mutations had occurred during *in vitro* passaging for selection of ciprofloxacin resistance, the entire genome of the CipR mutant was sequenced using high-throughput, single-molecule sequencing (GenBank: CP013853). This resulted in 113,394 polymerase reads with an average read length of 6,626 bp (126,205 subreads, avg. length of 5935 bp). The genome assembled into a single contig of 1,877,832 bp with 1787 CDS features, 10 rRNA genes and 38 tRNA genes. The assembly contained a single gap in the middle of one copy of the *Francisella* pathogenicity island [83]. This region is ~30 kb and nearly perfectly duplicated in *F*. *tularensis* Schu S4. However, both the full copy and partial copy of this region in our assembly are identical to the homologous regions in the parental strain.

In total, we identified 15 mutations in the CipR mutant genome compared to *F*. *tularensis* Schu S4 (GenBank: NC_006570) (Table 3). These included the three previously identified mutations in *gyrA* and *parE*, four mutations in intergenic regions (1 SNP and 3 indels) and eight additional mutations spread across seven different protein coding genes (Table 3). Most of the coding mutations were single nucleotide polymorphisms (SNPs) leading to amino acid substitutions in *fabH*, *fabF*, *FTT_0807*, *FTT_0676*, and *FTT_1573*. The *fupA* gene experienced a single base pair deletion at nucleotide 105 followed by a single base pair insertion at nucleotide 111, which maintained the reading frame of the gene (Table 3). Additionally, we identified a frameshift mutation caused by the addition of an “A” at nucleotide 174 to *FTT_0788c*/ *kdsD* (984 bp) (Table 3). KdsD catalyzes the conversion of the pentose pathway intermediate R5P into A5P. A5P is a precursor of KDO, an integral part of the LPS which is an established virulence factor for *F*. *tularensis* pathogenesis [63–68].

**Table 3:** Genetic alterations identified in the CipR strain of *F*. *tularensis*.

**Protein**	**Gene**	**Function**	**Gene Size**	Mutation and consequence*
YP_169795	*kdsD*	Isomerization of Ru5P to A5P.	987 bp	Addition of A at 174 bp.
YP_170322.1	*fabH*	3-oxoacyl-ACP synthase	972 bp	C→T at 805 bp. Pro→Ser
YP_169814.2	*capA*	Hypothetical poly-gamma-glutamate system protein	1,576 bp	A→G at 2721 bp. Asp→Gly
YP_170326.1	*fabF*	beta-ketoacyl-acylcarrier-protein synthase II	1,638 bp	A→G at 934 bp. Ser→Gly
YP_169692.1	*Ftt0676*	conserved hypothetical membrane protein	1,260 bp	A→G at 848 bp. Glu→Gly
YP_169915.1	*fupA*	Utilize iron bound to siderophores and for siderophore-independent iron acquisition	1,728 bp	Deletion of G at 105 bp; addition of G at 111 bp. Pro →Leu
YP_170495.1	*ftaG*	Hypothetical/ Surface antigen variable number repeat	2,379 bp	C→T at 1517 bp. Thr→Ile.
**Intergenic region**		**Function**		**Mutation**
*FTT_0025c*-*FTT_0026c*		Hypothetical protein & drug resistance transporter, Bcr/CflA subfamily		A→G
*glgC—glgA*		Glucose-1-phosphate adenylyltransferase & glycogen synthase		Deletion of A
*FTT_0517 –prmA*		Hypothetical protein & 50S ribosomal protein L11 methyltransferase		Deletion of TTTATATAAGT
*FTT_1486c –coaE*		Hypothetical protein & dephospho-CoA kinase		Deletion of A

* Bp numbers corresponds to ATG = 1.

### Construction of arabinose phosphate isomerase mutants

In order to explore the potential role of *kdsD* in virulence, a mutant in *kdsD* was constructed in a Schu S4 background. We used a modified TargeTron mutagenesis system and the Targetron plasmid pKEK1140 (Table 1) to disrupt the *kdsD* gene at site 611|612s using retargeted mobile group II introns as described previously [70]. Confirmation of insertion of the intron was demonstrated by PCR analysis using the primers that flanked the insert region. For DNA from the Schu S4 strain, a PCR fragment of ~1.3 kb was observed. However for mutant strains that contained the intron insert, a PCR fragment increased by approximately 900 bp was observed (data not shown).

As *F*. *novicida* is used as a surrogate for tularemia studies under BSL-2 conditions and to further verify the observations made with the *kdsD* Schu S4 mutant, we also examined a mutant for the gene encoding arabinose phosphate isomerase in the U112 strain from a previously constructed transposon library [69]. The homologous gene in *F*. *novicida* strain U112 is designated as *kpsF* (*FTN_1222*) [84] which we utilize here to distinguish between the two *Francisella* species and mutant strains. The *F*. *novicida kpsF* gene is 969 bp in length and 99% identical to the *F*. *tularensis kdsD* gene at the amino acid level. Two independent transposon mutants were identified having insertions in the *kpsF* gene; one was at nucleotide position 257 and the other was at position 394 [69]. However, we were unable to culture the latter mutant under various growth conditions, therefore all work described here was obtained using the former transposon mutant (BEI catalog # NR-6746).

### MIC and *in vitro* susceptibility testing of the *kdsD*/ *kpsF* mutants

To determine if the alteration of *kdsD* in the CipR mutant had any role in antibiotic resistance, MIC values were obtained for the *kdsD*::*ltrB*_*L1*_ mutant and compared to the Schu S4 parent and CipR mutant (Table 4). As expected, no difference in resistance to ciprofloxacin was observed between Schu S4 parent and *kdsD*::*ltrB*_*L1*_ (MIC = <0.03 μg/ml). In contrast, high levels of resistance were still detected for the CipR mutant (64 μg/ml) (Table 4) as previously reported [26]. Likewise, no resistance to ciprofloxacin was observed between the *F*. *novicida* U112 parent and the *kpsF*::T20 mutant (Table 4).

**Table 4:** MIC analysis.

Strain	Ciprofloxacin (μg/ ml)
*F*. *tularensis* Schu S4	<0.03
*F*. *tularensis* CipR	64
*F*. *tularensis kdsD*::*ltrB*_*L1*_	<0.03
*F*. *novicida* U112	<0.03
*F*. *novicida kpsF*::T20	<0.03
**QC Standards**	
*E*. *coli* ATCC 25922	<0.03
*S*. *aureus* ATCC 29213	0.5
*P*. *aeruginosa* ATCC 27853	0.25

In addition, we examined the *Francisella* mutant strains to determine if inactivation of *kdsD* or *kpsF* led to increased sensitivity to a panel of hydrophobic agents. As shown in Table 5, both the CipR and *kdsD*::*ltrB*_*L1*_ mutant strains showed an increase in sensitivity to PMB, Tween 20, and SDS but not Triton-X 100. However, the only measurements that were significant were for Tween 20. When the *kdsD*::*ltrB*_*L1*_ mutant was complemented with a functional *kdsD* gene on a plasmid, the levels of resistance to these compounds were restored to the parent levels. The *F*. *novicida kpsF*::T20 mutant was also examined; however, the only inhibitors which the *kpsF*::T20 mutant showed a significant increased sensitivity were Tween 20 and SDS (Table 5). This sensitivity was restored by complementation.

**Table 5:** Susceptibilities of *F*. *tularensis* and *F*. *novicida* to hydrophobic agents.

Strain	PMB ± SD^1^	Tween 20 ± SD	Triton X ± SD	SDS ± SD
*Ft* Schu S4	10.0 **±** 0	18.1 **±** 1.06	35.2 **±** 3.01	34.1 **±** 8.44
*Ft* CipR	13.7**±**3.22	32.5 **±** 5.85*	35.2 **±** 4.27	36.97**±**6.51
*Ft kdsD*::*ltrB*_*L1*_	11.6 **±** 0.2.718	24.9 **±** 0.93*	39.1 **±** 0.98	52.20 **±** 1.57
*Ft kdsD* Comp	10.0 **±** 0	18.7 **±** 2.00	36.3 **±** 1.12	32.0 **±** 8.39
*Fn* U112	19.2 **±** 2.54	10.0 **±** 0	31.7 **±** 2.51	23.0 **±** 2.00
*Fn kpsF*::T20	20 **±** 0.89	19.7 **±** 1.96*	29.6 **±** 0.51	29.1 **±** 0.56*
*Fn kpsF* Comp	20.2 **±** 0.40	10.0 **±** 0	28.8 **±** 6.75	24.8 **±** 1.57

Sterile paper disks (10 mm in diameter) were saturated in polymyxin B at 10 mg/ ml, Tween 20 at 5%, Triton X-100 at 5%, or SDS at 100 mg/ ml, dried, and placed in triplicate onto separate agar plates. Sensitivity to each agent was assessed by measuring the diameter of the zone of growth inhibition around the disk. The results are in millimeters and the average of the multiple measurements of disks from three separate experiments.

Those inhibitors which displayed significant differences (*p*< 0.05) with the mutants strain as compared to measurements with the respective parent strain are indicated by *.

^1^ **±** SD (standard deviation)

### Exogenous A5P restores growth of the *kdsD*::*ltrB*_L1_ mutant in Chamberlain’s defined medium but not CipR

**Fig 1:**
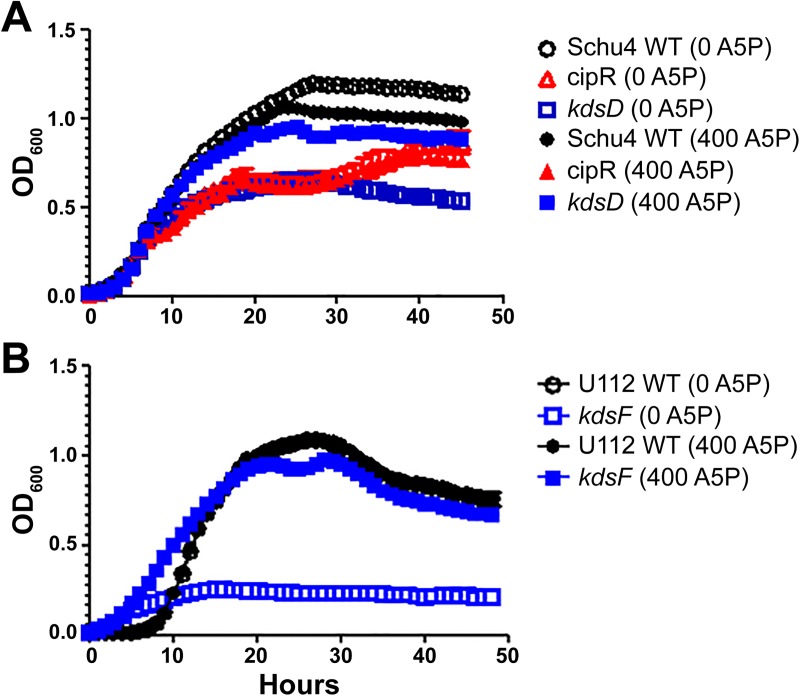
Growth assays. *F*. *tularensis* (A) or *F*. *novicida* (B) strains were grown in CDM at 37^°^C with (empty) or without (filled) the presence of A5P at a concentration of 400 μM. Growth was monitored by optical density. OD measurements were based upon quadruplicate samples and bars represent standard error of the mean. These data represent at least two separate experiments. The *F*. *tularenesis kdsD*::*ltrB*_*L1*_ and *F*. *novicida kpsF*::T20 mutants were severely altered for growth in Chamberlain’s medium. However, the addition of A5P to the medium significantly increased the growth of these strains. In contrast, the presence of A5P did not affect the growth of the *F*. *tularensis* CipR mutant as the two lines completely overlapped.

Additional characterization of the strains involved growth analysis in Chamberlain’s defined broth medium (CDM). As shown in Fig 1A, growth of the CipR and *kdsD*::*ltrB*_*L1*_ mutant strains versus Schu S4 in CDM showed significant differences in maximum density (*p* <0.0001), lag time (*p* <0.0001), and maximum growth rate (*p* <0.0001), respectively. For the *kdsD*::*ltrB*_*L1*_ mutant strain, we demonstrated that this growth defect in CDM was due specifically to the inactivation of the gene because complementation with a functional *kdsD* gene *in trans* on a plasmid completely restored growth to those of Schu S4 (S1 Fig).

Similar results for growth in CDM with the *F*. *novicida* U112 parent and *kpsF*::T20 mutant strains were observed (Fig 1B). Overall, the mutant was more impaired for growth and significant differences between the two strains were observed for maximum density (*p =* 0.0001), lag time (*p* <0.0001), and maximum growth rate (*p* <0.0001). Complementation with a functional gene was again able to restore growth of the mutant to wild-type levels (S1 Fig).

As the KdsD/ KpsF enzyme catalyzes the conversion of R5P into A5P, we hypothesized that these growth defects could be restored by adding A5P to the media. As shown in Fig 1, a significant increase in growth (as measured by maximum density, lag time, and growth rate; *p* <0.0001) was observed for both *F*. *tularensis kdsD*::*ltrB*_*L1*_ and *F*. *novicida kpsF*::T20 mutants when grown in the presence of 400 μM A5P versus growth without the additional A5P. In contrast, no difference was observed for the respective parent strains when grown with or without additional A5P (Fig 1). Interestingly, the Schu S4 CipR mutant did not show a significant increase in growth with the addition of A5P, despite the fact that it also contains a frameshift mutation in *kdsD*. Therefore, other mutations are presumably contributing to the growth defect in the CipR mutant.

### The CipR and *kdsD*::*ltrB*_*L1*_ mutants are affected in O-antigen expression of the LPS and capsule but not lipid A

**Fig 2:**
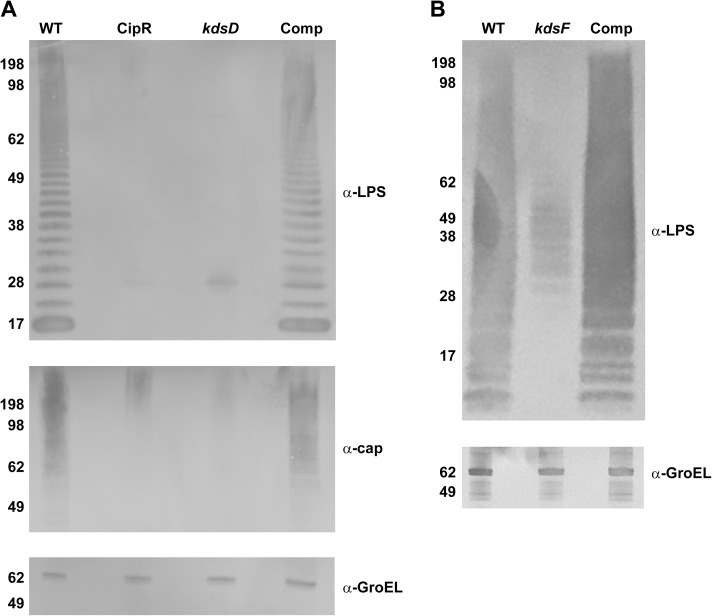
Western blot analysis of *Francisella* strains. Pellets of the (A) *F*. *tularensis* strains: Schu S4 (WT), CipR, *kdsD*::*ltrB*_*L1*_, and *kdsD* complement (Comp) or (B) *F*. *novicida* strains: U112 (WT), *kpsF*::T20, and *kpsF* complement (Comp) were lysed. Extracts were run on SDS-PAGE gels at equal concentrations and blotted with various antibodies as indicated: monoclonal antibody to the O-antigen of LPS of *F*. *tularensis* or *F*. *novicida*; monoclonal antibody to the O-antigen of the *F*. *tularensis* capsule; or a polyclonal antibody to GroEL of both *F*. *tularensis* and *F*. *novicida*. Molecular masses are indicated on the left in KDa. A) The LPS and capsule profiles of the CipR and *kdsD*::*ltrB*_*L1*_ mutants were defective in comparison to the WT strain. However, these profiles were restored for the *kdsD* mutant when complemented with a functional gene on a plasmid. Equal loading of sample material was demonstrated when blotting the extracts with an antibody directed against the GroEL protein. B) The LPS profile of the *kpsF*::T20 mutant was defective in comparison to the U112 parent strain. However, the profile was restored for the *kpsF*::T20 mutant when complemented with a functional *kdsD* from *F*. *tularensis* was provided on a plasmid. Equal loading of sample material was demonstrated when blotting the extracts with an antibody directed against the GroEL protein.

When performing western blot analysis with lysate material of equivalent bacterial CFU numbers extracted from wild-type Schu S4, CipR, or *kdsD*::*ltrB*_*L1*_ and monoclonal antibodies generated against LPS or the O-antigen capsule, the characteristic profiles of the wild-type strain were not observed in the CipR and *kdsD*::*ltrB*_*L1*_ mutant strains (Fig 2A). We note the extracts for the mutant strains did retain a slight band at approximately the 28 kDa marker. Likewise, when examining for the reactivity with the antibody to O-antigen capsule, some slight reactivity was still observed in the upper region (> 198 kDa) for the mutant strains. However, for both blots the level of reactivity was greatly diminished as compared to the parent strain. In contrast, no difference in GroEL levels was noted between the different strains. When the *kdsD* mutant strain was complemented *in trans* with a functional *kdsD* gene, the LPS and O-antigen capsule profiles were completely restored to this strain (Fig 2A). Similar results were observed by western analysis with the *kpsF*::T20 transposon mutant strain and monoclonal antibody directed against the LPS of *F*. *novicida* (Fig 2B), though some low level of reactivity with the LPS antibody was still detected. Again, the LPS profile could be completely restored via complementation (Fig 2B).

**Fig 3:**
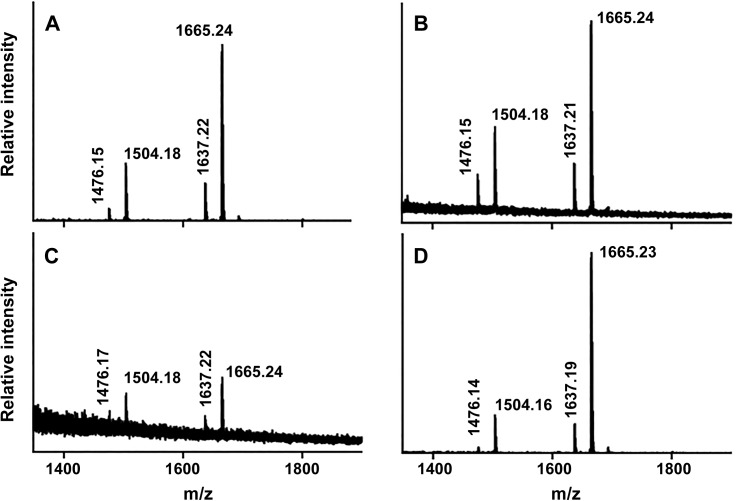
Characterization of the *F*. *tularensis* lipid A structure by MALDI-TOF mass spectrometry. LPS extracts from wild type Schu S4 (A), CipR (B), *kdsD*::*ltrB*_*L1*_, (C) and complemented *kdsD* mutant were analyzed by negative ion mode MALDI-TOF mass spectrometry. Monoisotopic mass/charge values of the four most prominent species within each spectrum are reported; these values correspond with the expected molecular weights of *F*. *tularensis* lipid A and its known variants as previously reported [29].

Negative ion mode MALDI-TOF mass spectrometry was used to further compare mutant and wild type LPS structures of *F*. *tularensis*. The MALDI process is capable of fragmenting the glycosidic bond that connects the core oligosaccharide to the lipid A moiety [85], allowing this structure to be elucidated from intact LPS preparations. We observed the prompt fragmentation of LPS and the resultant lipid A species using the data acquisition parameters used in these experiments (Fig 3). We observed the same lipid A structure and variants as reported by Kanistanon et al [29] where the species at m/z 1665.24 corresponds to the intact lipid A structure shown in Fig 3. The theoretical m/z for this molecule is 1665.25 [M-H]-. We determined experimental m/z values of 1665.24, 1665.24, 1665.24 and 1665.23 for LPS preparations from cultures of wild type Schu S4, CipR, *kdsD*::*ltrB*_*L1*_, and *kdsD* complemented strains, respectively. The species at m/z 1504.2 (delta 161.0 Da) corresponds to the intact lipid A structure minus one galactosamine unit. The minor peaks at m/z 1637.2 and 1476.2 correspond to shorter acyl chain lipid A variants (delta 28.0 Da) of the major peaks described above. We observed these same species in all four LPS preparations suggesting that the loss of the KDO structure in the two mutant strains (CipR and *kdsD*::*ltrB*_*L1*_) does not impact the structure of lipid A. We did observe a lower intensity of lipid A within the *kdsD*::*ltrB*_*L1*_ mutant sample which likely resulted from decreased extraction efficiency of the sample material between the solvents during preparation of an LPS mutant. However, the main goal of this experiment was to simply demonstrate that the loss of KDO did not affect the lipid A structure in the remainder of the LPS molecule for the mutant strains which these results do conclusively show.

### Interaction of *Francisella* strains with macrophage-like cells

**Fig 4:**
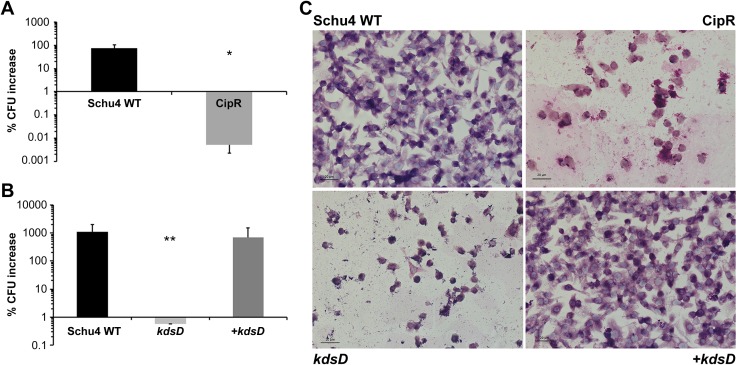
The interaction of *F*. *tularensis* CipR and *kdsD*::*ltrB*_*L1*_ mutants with macrophage-like cells show a decrease in CFU recovery and disruption of the host monolayer. J774A.1 cells were infected with A) Schu4 WT or CipR or B) Schu4 WT, *kdsD*::*ltrB*_*L1*_ mutant, or complemented *kdsD* mutant (+ *kdsD*) at an MOI of ~100:1. Data depict the difference in the percentage of viable CFU counts measured at 4h and 24 h post-challenge following gentamycin protection assays. * *p*<0.0001 or ** *p*<0.004 as compared to the parent. Error bars represent standard deviation from three independent experiments. Panel C shows coverslips of monolayers of J774A.1 cells infected with *F*. *tularensis* Schu S4 (WT), CipR, *kdsD*::*ltrB*_*L1*_, or complemented *kdsD* mutant (*+kdsD*). Cells were fixed at 24 h post-infection and then subjected to Wright Giemsa staining. Scale bar = 20 μm.

**Fig 5:**
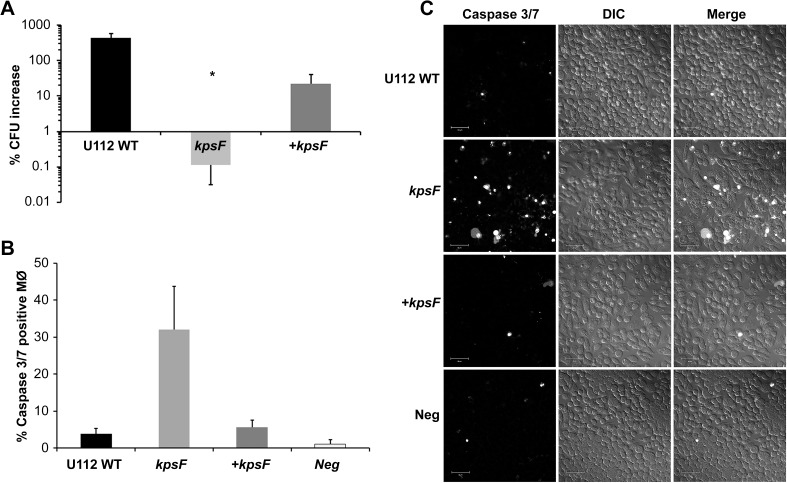
The interaction of *F*. *novicida kpsF*::T20 mutant with macrophage-like cells showed a decrease in CFU recovery and the host cell monolayer undergoing cell death. J774A.1 cells were infected with A) the *F*. *novicida* U112 parent strain, *kpsF*::T20 mutant, or complemented *kpsF* mutant (+ *kpsF*) at a MOI of ~100:1. Data depict the difference in the percentage of viable CFU counts measured at 4h and 24 h post-challenge following gentamycin protection assays. * *p* <0.0001 as compared to the parent. Error bars represent standard deviation from the average differences from three independent experiments. B) J774A.1 cells, seeded at the same density on coverslips, were left uninfected or infected with *F*. *novicida* U112, *kpsF*::T20, or the complemented mutant. At 18 h post-infection, cells were incubated with Caspase 3/7 and infected J774A.1 cells were counted to determine the percentage of cells fluorescing due to cell death. The total number of cells counted for each of the samples was WT infected = 1,596; *kpsF*::T20 infected = 621; + *kpsF* infected = 892 cells; and uninfected negative control = 689. C) Images of the cells incubated with Caspase 3/7 green ready probes imaged live and fluorescing (left panel) with an accompanying differential interference contrast (DIC; middle column) and merge (right column) images taken to show cell density. Cells infected with *kpsF*::T20 mutant showed an increase in fluorescent signal in the monolayer indicating cells destined for cell death, in contrast to uninfected cells and those infected with WT or + *kpsF*. Scale bar = 50μm.

The fate of the *F*. *tularensis* strains following uptake by J774A.1 cells was studied using a gentamycin protection assay to examine CFU recovery at 4 h and 24 h post-challenge. These data are depicted in Figs 4A, 4B and 5A as “percent CFU increase” and show the differences in CFU recovery between these time points for the various *F*. *tularensis* and *F*. *novicida* strains. Little difference in the initial recovery of CFUs between Schu S4 and CipR or *kdsD*::*ltrB*_*L1*_ strains was observed at the 4 hour time point (data not shown), suggesting the initial uptake of the bacteria was not affected. However, after a 24 h incubation period, the recovered number of CFUs had increased by several logs for J774A.1 cells infected with Schu S4. In contrast, the number of CFUs recovered from J774A.1 cells infected with CipR (Fig 4A) or *kdsD*::*ltrB*_*L1*_ (Fig 4B) mutant strains had decreased significantly as compared to CFU counts with the wild-type strain. To demonstrate if this difference was due specifically to the inactivation of *kdsD*, J774A.1 cells were infected with the *kdsD* complemented strain. As shown in Fig 4B, a several log increase of CFUs was observed for macrophages infected with the complemented strain after the 24 h post-challenge time point.

During these studies, a disruption in the confluence of the macrophage monolayers was also noted after the 24 h incubation with either CipR or *kdsD*::*ltrB*_*L1*_ *F*. *tularensis* mutant strains but not with the parent Schu S4 or *kdsD* complemented strains (Fig 4C). Previous studies with other *F*. *tularensis* mutants containing LPS defects had shown similar induction of macrophage death [33, 86, 87]. Therefore, the loss of CFU recovery with the *F*. *tularensis* mutants from our current study could be due to either a defect in the ability of the mutants to replicate intracellularly or the loss of the host cells and therefore the intracellular replicative niche of the bacteria.

Similar results were observed when examining the recovery of CFUs with the *F*. *novicida kpsF*::T20 mutant from infected J774A.1 cells. As shown in Fig 5A, a several log CFU increase was observed with the U112 wild-type strain 24 h-post challenge. In contrast, no increase in the CFUs was observed with the *kpsF*::T20 mutant and significantly differed (*p* = 0.0034) from J774A.1 cells infected with the wild-type strain. However, when a functional gene was supplied to the mutant via complementation, CFU recovery was restored to the mutant strain. The fate of the macrophages infected with the *F*. *novicida* LPS mutant was also examined. We demonstrated that cell death was occurring at 18 h post infection as detected by Caspase 3/7 activity (Fig 5B & 5C), and the increase in Caspase 3/7 activity can be seen as early as 12 h post infection (data not shown). The J774A.1 cells infected with the *kpsF*::T20 mutant strain were found to be undergoing host cell death at a much higher level than observed with cells infected with the U112 strain or uninfected macrophages. However, if the mutant was complemented, little cell death was observed (Fig 5B & 5C).

### The CipR and *kdsD*::*ltrB*_*L1*_ mutants of *F*. *tularensis* were highly attenuated in mice

**Fig 6:**
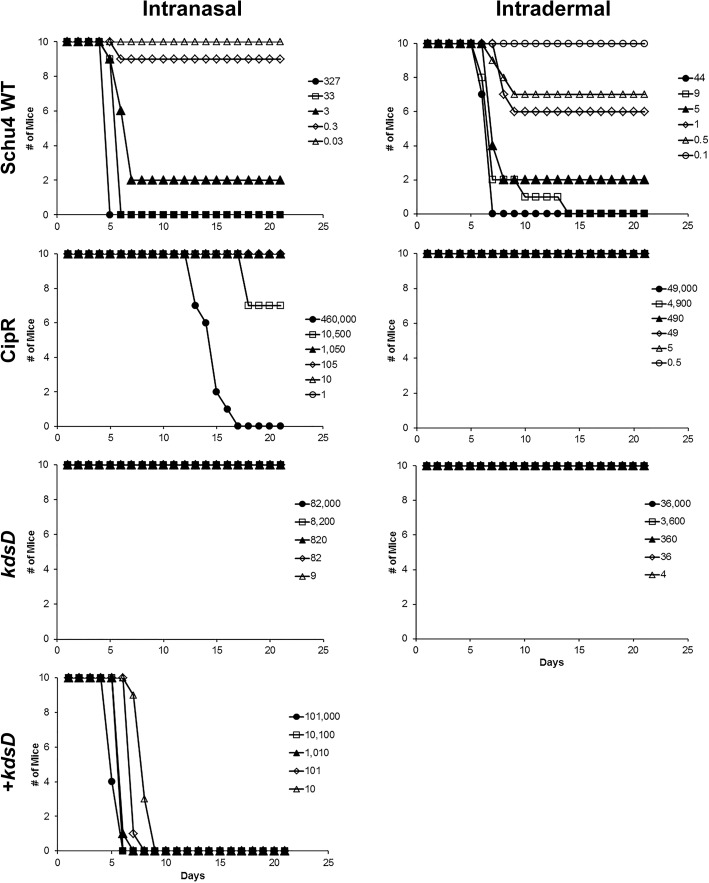
The CipR and *kdsD*::*ltrB*_*L1*_ mutant strains of *F*. *tularensis* were attenuated in BALB/c mice by intranasal and intradermal challenge. Groups of BALB/c mice (n = 10) were challenged and survival monitored following infection by intranasal or intradermal injections using the wild-type Schu S4 strain, CipR mutant, *kdsD*::*ltrB*_*L1*_ mutant, and the complemented *kdsD* mutant strain, as indicated. The calculated LD_50_ values from these experiments are included in Table 6.

To determine if the CipR and *kdsD*::*ltrB*_*L1*_mutant strains were still virulent in mice, various models of tularemia challenges were tested. The murine LD_50_ measurements for the wild-type strain by intranasal and intradermal challenges were both determined to be 1–2 CFU (Fig 6; Table 6). In contrast, the LD_50_ values for the CipR mutant by these same challenge routes were greatly increased: 14,000 and >49,000 CFU, respectively (Fig 6; Table 6).

**Table 6:** Calculated LD_50_ for the *F*. *tularensis* and *F*. *novicida* wild-type and mutant strains.

Strain	LD_50_ Intranasal	LD_50_ Intradermal
*F*. *tularensis*		
Schu S4	1–2 CFU	1–2 CFU
CipR	14,468 CFU	>49,000 CFU
*kdsD*::*ltrB*_*L1*_	>82,000 CFU	>36,000 CFU
*kdsD* complement	<10 CFU	ND
*F*. *novicida*		
U112	<23 CFU	ND
*kpsF*::T20	25,119 CFU	ND
*kpsF* complement	32 CFU	ND

Likewise, complete attenuation was observed for mice challenged by the intranasal and intradermal routes for all challenge doses with the *kdsD*::*ltrB*_*L1*_ mutant. LD_50_ measurements for the *kdsD*::*ltrB*_*L1*_ mutant were >82,000 and >36,000 CFU, respectively (Fig 6; Table 6). To demonstrate this severe attenuation of the *kdsD*::*ltrB*_*L1*_ mutant was due specifically to the inactivation of the *kdsD* gene, mice were intranasally challenged with the complemented mutant (Fig 6; Table 6). Almost complete restoration of virulence was observed when mice were challenged by the intranasal route with a complemented *kdsD* strain, with the LD_50_ determined to be <10 CFU (Table 6).

### The recovery and dissemination of the CipR and *kdsD*::*ltrB*_*L1*_ mutants were hindered after intranasal challenge

**Fig 7:**
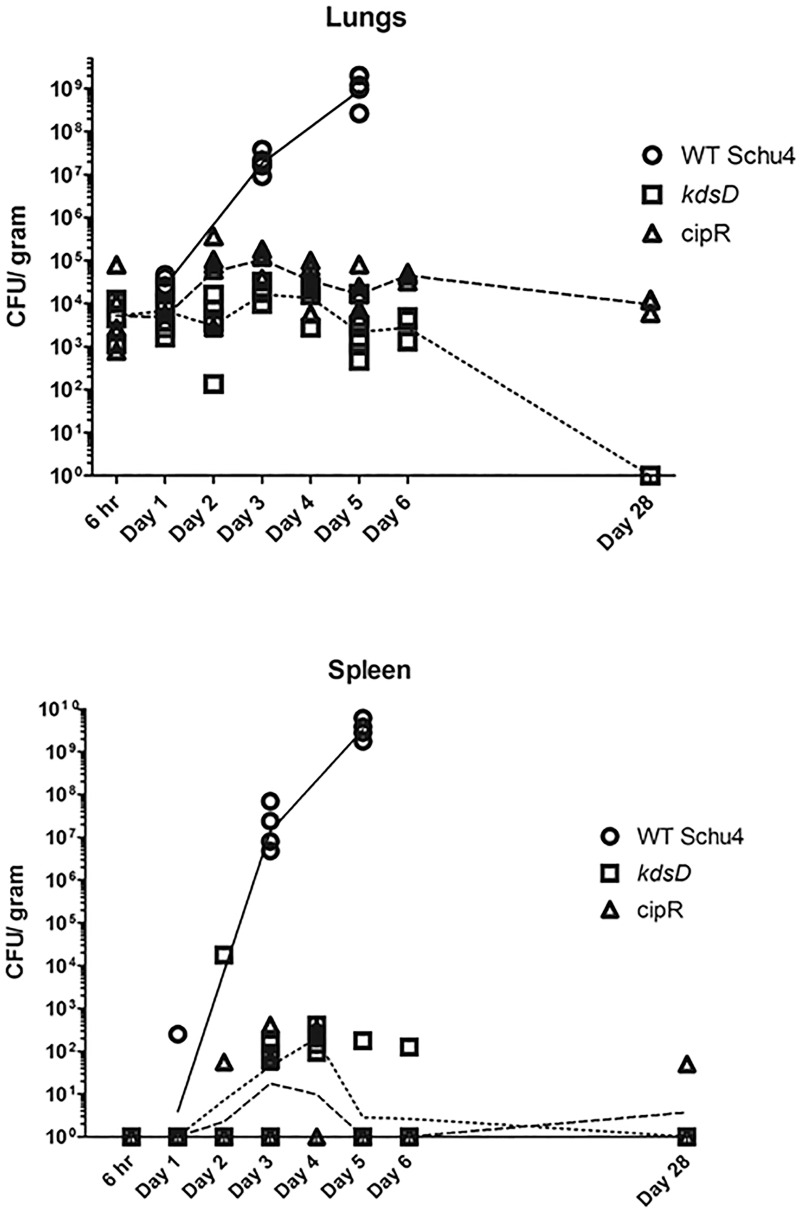
Dissemination studies of mice challenged intranasally with *F*. *tularensis*. From a single challenge study, mice were exposed intranasally with Schu S4 (131 CFU), CipR mutant (1,750 CFU), or *kdsD*::*ltrB*_*L1*_ (6,000 CFU). At set time points, mice were euthanized, and the lungs and spleens were harvested. The lungs and spleens, as indicated, were homogenized and plated to determine bacterial recovery. For each time point, five mice were assayed, except for day 5 for wild-type challenged mice and Day 28 for the CipR mutant challenged mice due to mice having succumbed to infection. The lines (solid = WT Schu S4, hashed = CipR mutant, and dotted = *kdsD*::*ltrB*_*L1*_ are connecting at the geometrical means at the data points of CFU recovery from the respective organs and represent the overall trend during the course of infection.

**Fig 8:**
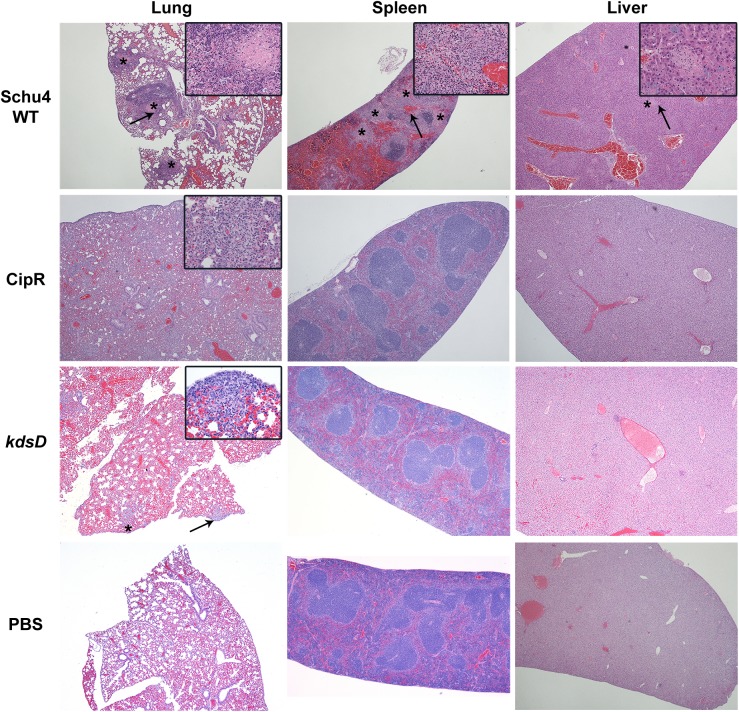
Pathology of mice challenged intranasally with *F*. *tularensis* 5 days post-challenge. Mice were challenged intranasally with Schu S4 WT (131 CFU), CipR mutant (1,750 CFU), or *kdsD*::*ltrB*_*L1*_ (6,000 CFU). Also included as a control for these studies were mice receiving PBS alone by intranasal administration. The strains used for challenge and HE stained organ (lung, spleen, and liver) are as indicated. All samples shown are at 5 days post-challenge when the Schu S4 challenged mice were moribund; in contrast, the mutant challenged mice displayed no clinical signs of infection at this time point. Schu S4 WT, Lung (4x)–multifocal areas of inflammation and necrosis (*); arrow indicates the inset area. Inset (40x)–necrosis admixed with inflammatory cells. Spleen (4x)–coalescing areas of necrosis (*) affecting red pulp and white pulp; arrow indicates the inset area. Inset (40x)–necrosis admixed with inflammatory cells. Liver (4x)–Single focus of necrosis (*); arrow indicates the inset area. Insert (40x)–necrosis with few inflammatory cells. CipR mutant, Lung (4x)–diffuse coalescing necrotic areas. Inset (40x)–necrosis admixed with inflammatory cells. Spleen (4x)–normal. Liver (4x)–normal. *kdsD*::*ltrB*_*L1*_, Lung (4x)–few foci of necrosis (*); arrow indicates the inset area. Inset (40x)–necrosis admixed with inflammatory cells extends to the surface of the lung. Spleen (4x)–normal. Liver (4x)–normal.

To determine the fate of the CipR and *kdsD*::*ltrB*_*L1*_ *F*. *tularensis* mutants after intranasal challenge, groups of mice were separately exposed to wild-type Schu S4 (131 CFU), CipR (1,750 CFU), or *kdsD*::*ltrB*_*L1*_ (6,000 CFU). At set time points after challenge, mice from each group were euthanized for determining either bacterial burden from lungs and spleens (Fig 7) or assessing histopathological changes (Fig 8).

For mice receiving the wild-type Schu S4, the number of bacteria recovered from the lungs and spleens increased exponentially after Day 1 (Fig 7). By Day 5, all mice were moribund and one had succumbed to infection. For the remaining mice, lungs and spleens contained approximately 10^9^ CFU/ g (Fig 7). In contrast, the number of CFUs recovered from the organs of mice infected with the CipR or the *kdsD*::*ltrB*_*L1*_ mutant strains were greatly reduced as compared to mice challenged with the Schu S4 parent strain. For the lungs over the first 7 days of testing, little change in the recovered CFUs was observed as compared to CFUs at 6 hours post-challenge. At the end of the study on Day 28, the remaining challenged mice were tested for the presence of bacteria within their organs. On Day 26, two of the CipR challenged mice had succumbed to infection. The lungs from the remaining CipR challenged mice were still shown to contain some CFUs but still at a relatively low level. All of the *kdsD*::*ltrB*_*L1*_ challenged mice survived until Day 28, and the lungs of these mice were found to be free of *F*. *tularensis* (Fig 7).

Overall very few of the spleens of the CipR or *kdsD*::*ltrB*_*L1*_ mutant challenged mice had any CFUs recovered over the first 2 days. Over the remaining week of testing, the number of spleens shown to contain CFUs was inconsistent, with many being negative for the presence of *F*. *tularensis*. At the end of the study, one of the spleens from the CipR challenged mice had a low level of CFUs recovered. However, none of the spleens for the *kdsD*::*ltrB*_*L1*_ challenged mice had bacteria present.

### Histopathologic analysis of intranasal challenged mice

Along with testing for the presence and trafficking of *F*. *tularensis* from the lungs, additional mice (n = 3) were processed to compare histopathological differences in disease progression following intranasal challenge with the wild type Schu S4 or the two mutant strains (Fig 8). All pathology images in Fig 8 are comparing the challenged mice at Day 5. It is at this time point that all wild-type challenged mice become moribund versus complete survival for the mice challenged with the CipR or *kdsD* mutant strains. Additional pathology images throughout the time course of this study are provided as supplementary data (S2–S4 Figs) as indicated below.

When examining organs from mice on Day 1 post-challenge with Schu S4, the mice did not have any lesions suggestive of tularemia. The lesions in these animals were limited to non-specific hyperplasia of lymphoid tissue in various lymph nodes and in the white pulp of the spleen (S2 Fig). However, by Day 3 post-exposure, mice demonstrated lesions consistent with tularemia infection [21, 23, 88]. These lesions consisted of small multifocal random areas of necrosis and neutrophilic inflammation in the sinusoids of the liver, red and white pulp of the spleen, cortex and medulla of the lymph nodes, and interstitium of the lung (S3 Fig). For mice on Day 5 post-exposure, lesions consisted of random foci of lytic necrosis and neutrophilic inflammation in the liver, spleen, lungs, and lymph nodes (Fig 8). Additionally, there were numerous intralesional large colonies of coccobacilli, morphology consistent with *F*. *tularensis*.

When examining mice receiving the CipR strain, no lesions were observed on Day 1 (S2 Fig). By Day 2 post-exposure, 2/3 mice had minimal multiple foci of necrosis and neutrophilic inflammation in the lung (pneumonia). The areas of necrosis and inflammation in the lung appeared to be random and associated with small airways (alveoli). Similar pneumonia is noted in all day 3 mice (S3 Fig). In all Day 4 mice, there is similar minimal to mild pneumonia with extension to the surface of the lungs (pleura), and admixed with the necrosis and neutrophils are discernible histiocytes/macrophages. In all Day 5 post-exposure mice, there was mild to moderate pneumonia with extension to the surface of the lungs (pleura) and admixed with the necrosis and neutrophils are discernible histiocytes/macrophages (Fig 8). The pneumonia in the Day 5 mice involved 10–25% of the lung parenchyma versus less than 10% of the lung parenchyma for Day 2 through Day 4 mice. In 1/3 mice from the Day 5 post-challenge, there was minimal focal necrosis with neutrophilic inflammation in the liver (Fig 8). At Day 28, there were diffuse mild to moderate to marked lymphoplasmacytic and histiocytic pleuropneumonia with some neutrophils associated with the aforementioned inflammatory infiltrate in the lung parenchyma and within bronchiolar lumina. There was minimal lymphoid hyperplasia in the white pulp of the spleen in all mice. Similar lymphoid hyperplasia was observed in 2/5 Day 28 mice in the tracheobronchial lymph node (S4 Fig). It should be noted that all of the *F*. *tularensis* challenged mice were compared to mice receiving only PBS intranasally for comparison, and no lesions were noted in any of the organs (Fig 8).

Overall, similar lesions were observed for mice challenged intranasally with the *kdsD*::*ltrB*_*L1*_ mutant as described above for the CipR mutant. Mice sacrificed on Day 1 demonstrated multiple and random foci of necrosis and neutrophilic inflammation in the lung (pneumonia) (S2 Fig). In all mice in this group, the severity was judged to be only minimal affecting less than 5% of the lung. All Day 2 mice had similar minimal pneumonia. By Day 3 post-infection, there was similar pneumonia, mild in two mice and moderate in one mouse. The necrosis and inflammation in the Day 3 mice extended to the pleural surface in 2/3 mice and affected pulmonary vessels in all three. The pneumonia was still characterized by necrosis and neutrophilic infiltrates, but in two mice, histiocytes/macrophages were also observed (S3 Fig). Within the Day 4 group, there was minimal to moderate pneumonia that extended to the pleural surface in all three mice. The pneumonia was characterized by necrosis and neutrophilic, histiocytic, and lymphoplasmacytic infiltrates. Additionally on Day 4, there are random areas of hepatocyte degeneration and necrosis with neutrophilic and histiocytic inflammation. In all Day 5mice, there was mild multifocal neutrophilic, histiocytic, and lymphoplasmacytic pneumonia and mild necrosis with neutrophils and histiocytes in the liver (Fig 8). On Day 28, 2/3 mice had mild multifocal lymphoplasmacytic and histiocytic inflammation in the lung and pleura with no necrosis (S4 Fig).

### The CipR and *kdsD*::*ltrB*_*L1*_ *F*. *tularensis* mutants are attenuated when mice are exposed to aerosolized bacteria

**Fig 9:**
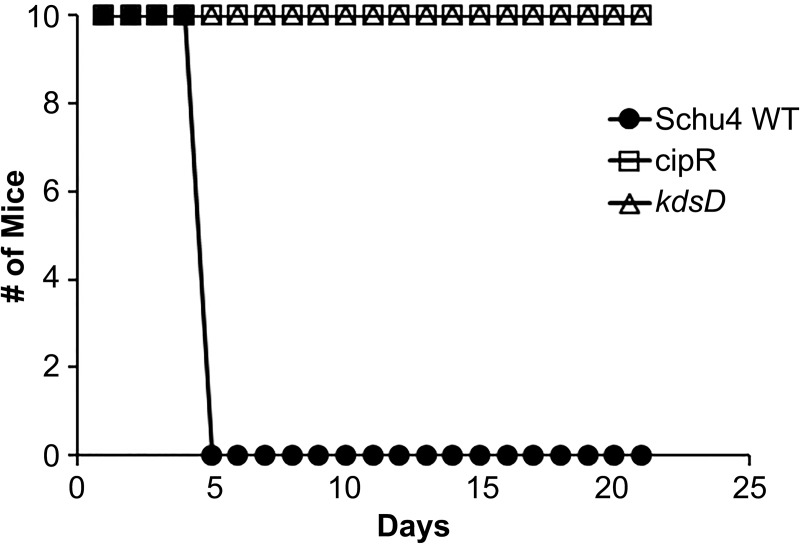
The CipR and *kdsD*::*ltrB*_*L1*_ mutant strains of *F*. *tularensis* are attenuated in BALB/c mice following small particle aerosol challenge. A control group of mice were exposed to 33 LD_50_ of the parent Schu S4 strain, and all succumbed or were moribund by Day 5. In contrast, other mice were challenged by aerosol with either the CipR strain (receiving the equivalent of 43 wild-type LD_50_) or the *kdsD*::*ltrB*_*L1*_ mutant (receiving the equivalent of 100 wild-type LD_50_) and all survived challenge to Day 21.

Aerosol exposure of *F*. *tularensis* is the greatest threat from a biodefense perspective. Accordingly, the ability of the CipR and *kdsD*::*ltrB*_*L1*_ mutant strains to be aerosolized and cause infection in mice was also explored. Since both mutant strains were highly attenuated via the intranasal route of challenge, mice were exposed to a single high dose of *F*. *tularensis* via small particle aerosol. All mice survived exposure to the CipR mutant (the equivalent of 43 wild-type LD_50_) or *kdsD*::*ltrB*_*L1*_ mutant (the equivalent of 100 wild-type LD_50_) during the entire course of the study (21 days) (Fig 9). In contrast, all mice receiving aerosolized Schu S4 (33 LD_50_) succumbed to infection by Day 5 (Fig 9). The LD_50_ for BALB/c mice exposed to aerosolized Schu S4 bacteria is approximately 300 CFU [21, 22].

To determine what, if any, pathological changes occurred in mice surviving aerosol challenge with the mutant strains as compared to mice exposed to the wild-type Schu S4 strain, mice were euthanized when moribund by Day 5 or following survival 21 days post challenge (CipR and *kdsD*::*ltrB*_*L1*_ mutant challenged mice) and processed for histopathologic examination.

**Fig 10:**
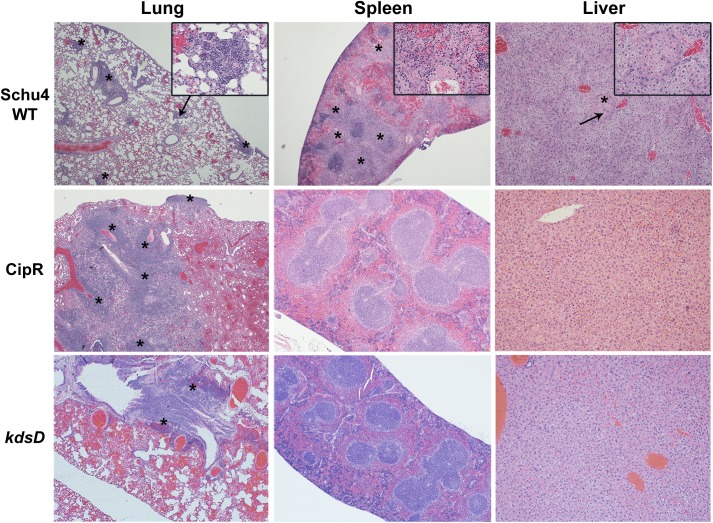
Pathology of mice challenged by small particle aerosol with *F*. *tularensis*. The strains used for aerosol challenge (Schu S4 WT, CipR, and *kdsD*::*ltrB*_*L1*_) and the HE stained organ (lung, spleen, and liver) are as indicated. The mice challenged with Schu S4 were collected at Day 5 when all mice had succumbed to infection. The mice challenged with the CipR or *kdsD*::*ltrB*_*L1*_ mutant survived till the end of the study (Day 21) and displayed no clinical signs of infection. Schu S4 WT: Lung (4x)–multifocal areas of inflammation and necrosis (*); arrow indicates the inset area. Inset (40x)–necrosis admixed with inflammatory cells. Spleen (4x)–diffuse coalescing areas of necrosis (*) affecting red pulp and white pulp. Inset (40x)–necrosis (*) admixed with inflammatory cells; arrow indicates the inset area. Liver (10x)–Single focus of necrosis (*); arrow indicates the inset area. Insert (40x)–necrosis with few inflammatory cells. CipR: Lung (4x)–Diffuse coalescing areas of necrosis (*) with inflammatory cells that extend to the surface of the lung. Spleen (4x)–normal. Liver (10x)–normal. *kdsD*::*ltrB*_*L1*_: Lung (4x)–foci of necrosis (*) centered on a large airway. Spleen (4x)–normal. Liver (10x)–normal.

For mice challenged with the wild-type Schu S4 strain, the microscopic lesions were typical of *F*. *tularensis* and resulted in the death of these mice. The most significant lesions were noted in the spleen and lung. The lesions in the spleens were characterized by necrosis with neutrophilic inflammation and fibrin (Fig 10). In the lungs, there was necrosis and neutrophilic inflammation (necrosuppurative) of the parenchyma and pleura (pleuropneumonia). There was also mild to moderate inflammation of lung vessels (vasculitis) characterized by necrosis and neutrophilic inflammation within vessel walls, often with fibrin thrombi (Fig 10). The livers of the moribund mice contained multiple minimal to mild areas of necrosis with neutrophilic inflammation and intracellular coccobacilli (Fig 10). Other lesions in the mice were typical of tularemia to include bone marrow necrosis, nasal turbinate/pharyngeal mucosal necrosis, lymph node necrosis, and/or fibrin thrombi in various organs (data not shown).

For mice challenged by aerosol with the CipR mutant, the only microscopic lesion of note in all mice examined was a lymphoplasmacytic and histiocytic pleuropneumonia with some neutrophils associated with the inflammatory infiltrate in the lung parenchyma and within bronchioles (Fig 10). This inflammation is more chronic with a minimal active component and suggests the lung inflammation may be resolving following *F*. *tularensis* infection. There is minimal lymphoid hyperplasia in the white pulp of the spleen which may be due to chronic antigenic stimulation from resolving *F*. *tularensis* infection (Fig 10). Likewise for mice surviving aerosol challenge with the *kdsD*::*ltrB*_*L1*_ mutant, a very similar course of disease for the mice was observed as described above for the CipR mutant strain. The one slight difference was that the inflammation in the lungs of the mice exposed to the CipR mutant may be slightly more severe based upon subjective assessment of the amount of lung parenchyma affected. Overall for the lungs of the *kdsD*::*ltrB*_*L1*_ exposed mice, there was minimal to mild lymphoplasmacytic and histiocytic inflammation with few scattered neutrophils, often associated with bronchioles in the lung (Fig 10). The inflammation is indicative of a chronic resolving process from aerosol exposure to the *kdsD*::*ltrB*_*L1*_ mutant. Finally, no other lesions or pathologic changes were noted in the liver or spleens of the mice surviving aerosol challenge with CipR or *kdsD*::*ltrB*_*L1*_ mutant in contrast to the severe pathologic changes observed with mice succumbing to infection with the wild-type Schu S4 strain (Fig 10).

### The *kpsF*::T20 mutant of *F*. *novicida* is highly attenuated for mice

**Fig 11:**
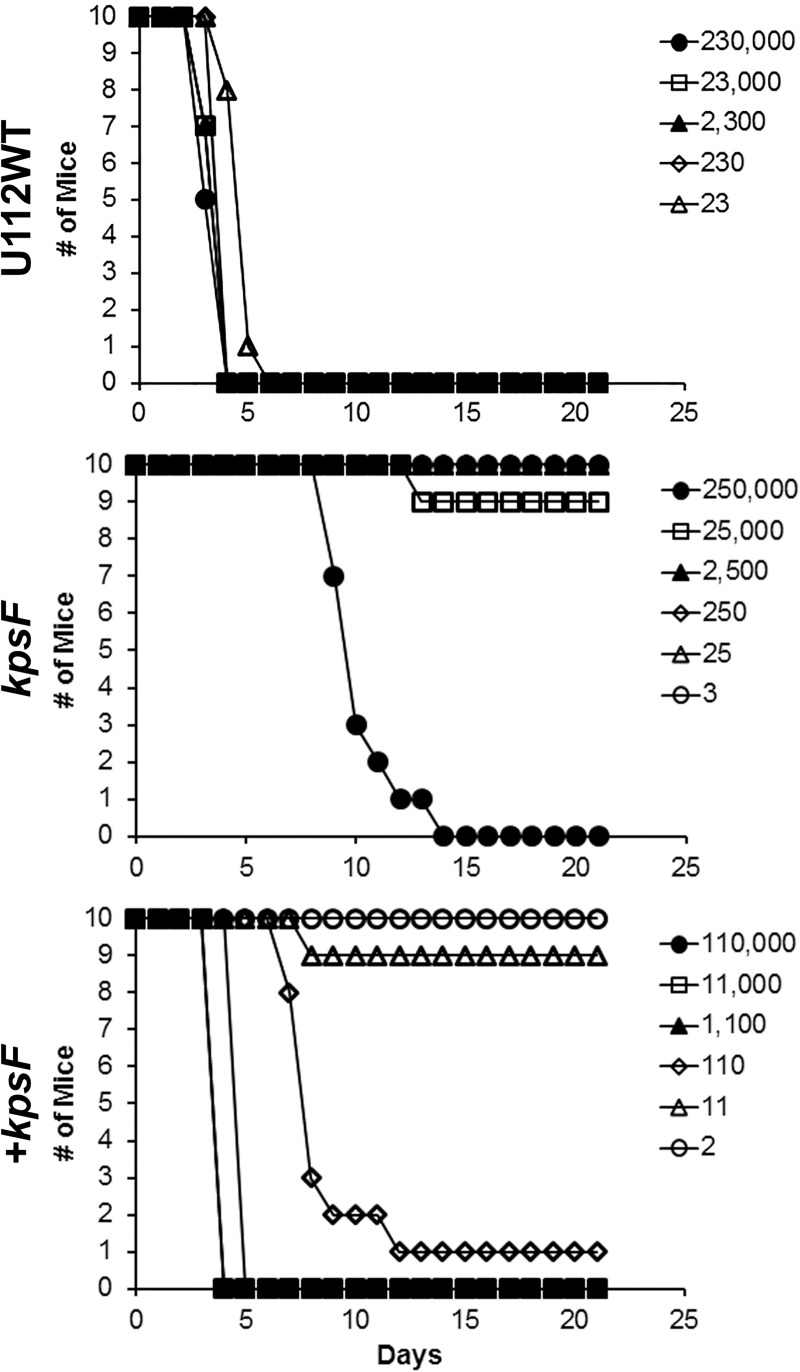
The *kpsF*::T20 mutant of *F*. *novicida* is highly attenuated by intranasal challenge in BALB/c mice. Groups of BALB/c mice were challenged intranasally with the parent U112 strain of *F*. *novicida*, the *kpsF*::T20 mutant, or the complemented *kpsF* mutant. The calculated LD_50_ values from these experiments are included in Table 6.

As *F*. *novicida* is frequently used as a surrogate for tularemia studies, we wished to demonstrate if the *F*. *novicida kpsF*::T20 mutant was also attenuated in a murine model of inhalational tularemia to further corroborate the studies performed with the *kdsD* mutant in *F*. *tularensis* described above. As shown in Fig 11 and Table 6, we challenged groups of mice intranasally with either the *F*. *novicida* U112 parent strain or the *kpsF*::T20 mutant. All mice receiving the parent strain succumbed to infection, and the LD_50_ was >24 CFU (Fig 11 & Table 6). However, mice receiving the *kpsF*::T20 mutant were able to survive challenge except for those receiving the highest mutant doses (Fig 11). The LD_50_ for the *kpsF*::T20 mutant was calculated to be 25,119 CFU (Table 6). The attenuation observed for the *kpsF*::T20 mutant nearly restored when a functional gene was supplied *in trans* on a plasmid (Fig 11). The LD_50_ for the complemented strain was calculated to be 32 CFU (Table 6).

## Discussion

Bacterial resistance to antibiotics is a serious threat to the public health and the biodefense communities. The goal of this study was to further characterize a ciprofloxacin resistant strain of *F*. *tularensis* [26]. Our intent was to better understand the bacterial pathogenesis and threat a resistant strain might pose whether it arise naturally or made intentionally. The major findings of our work are as follows. 1) The CipR mutant was severely hampered in its ability to cause infection in all tested murine models of tularemia. 2) In addition to the previously identified changes to *gyrA* and *parE* [26], the genome of the CipR mutant contained additional genetic alterations, including in *kdsD*, the mutated gene that likely is responsible for the attenuation in the CipR mutant strain. 3) Both *F*. *tularensis* Schu S4 *kdsD* and *F*. *novicida* U112 *kpsF* mutations led to LPS alterations and attenuation in mice.

In addition to being highly resistant to ciprofloxacin, the CipR mutant displayed other phenotypes: *in vitro* growth defects, increased sensitivity to hydrophobic agents, LPS profile alterations, increased induction of mammalian cell death, and high attenuation in multiple models of murine tularemia. Many of these additional characteristics, albeit not all, are likely due to the frameshift in *kdsD* which occurred during the selection process but was not associated with ciprofloxacin resistance. Mutations specifically to the *kdsD*/ *kpsF* gene replicated many of the characterizations observed with the CipR mutant. We focused on the mutation of *kdsD* in the CipR mutant to better understand the phenotype of this strain since it was the only gene, outside of *parE*, exhibiting a frameshift mutation which presumably would result in a higher impact effect. We hypothesized that KdsD plays an important role in virulence because it synthesizes an LPS precursor and LPS is an established virulence factor for *F*. *tularensis* [63–68].

Whereas *kdsD* and *parE* are frameshift mutations, the other altered genes on the chromosome of the CipR mutant led to amino acid substitutions within the effected protein (Table 3). This does not discount the possibility that these other mutations may contribute in part to the loss of virulence or other phenotypes observed for the CipR strain. Some of these alterations occurred to genes encoding for other known *Francisella* virulence factors, such as *Ftt_0807*/ *capA* [89, 90] and *fupA* [91, 92]. The role, if any, in *Francisella* pathogenesis for the other proteins remains to be determined. The FabH and FabF proteins are both enzymes involved in type II fatty acid biosynthesis system which are required for synthesis of essential lipoproteins, phospholipids, and LPS [93, 94]. Presently, little else is known about FtaG (FTT_1573c), an outer membrane surface antigen [95]. Likewise, *Ftt0_676* encodes for an ion transporter which has been shown to be downregulated when *F*. *tularensis* is present within macrophages [96].

In addition, as shown in Fig 1A, the growth defects of the *kdsD* mutant in Schu S4 and *kpsF*::T20 mutant in U112 were restored to the respective wild-type levels when providing exogenous A5P, the end product of the _D_- arabinose 5-phosphate isomerase activity. This growth defect was not able to be restored to the CipR strain when providing A5P, demonstrating additional defects to this mutant.

Many of the observed characteristics for CipR mutant were similar to the Schu S4 *kdsD*::*ltrB*_*L1*_ mutant, such as a defect in the LPS profile. This would not be surprising as the *kdsD* gene encodes for the enzyme catalyzing the first step for KDO biosynthesis. As the CipR and *kdsD*::*ltrB*_*L1*_ mutants would not be able to form KDO, the link between lipid A and the polysaccharide core and O-antigen would be affected, therefore the resulting mutant strains would be severely disrupted for these structures. Although, our study is the first to examine a *kdsD* mutant of *F*. *tularensis*, a recent report describes a transposon mutant in *kdsB* (*FTT_1478c*) which encodes for cytidine 5'-monophospho-KDO synthase. The *kdsB* mutant also did not react with antibodies generated against LPS or the O-antigen capsule, but virulence studies were not reported for this mutant [86]. The results for a defect of the capsule on both the CipR and *kdsD*::*ltrB*_*L1*_ mutants provide further evidence that a full-length LPS serves a scaffold for formation of this structure as previously suggested [66]. However, a previous study with a *lpxL* (encoding for a lipid A acyltransferase) mutant which expresses only a portion of the LPS core still expressed a capsular antigen [63]. The exact reason for this discrepancy remains to be determined. In addition to defects in the LPS profile, the CipR and *kdsD*::*ltrB*_*L1*_ mutants also recapitulate characteristics similar to phenotypes described for other *F*. *tularensis* LPS and O-antigen mutants: early induction of death to macrophages [86, 87] and attenuation in murine models of tularemia [66, 97, 98].

In our current study, the pathology noted with mice challenged intranasally or by small particle aerosol with the wild-type Schu S4 are in general agreement with other published studies [21, 88]. For pneumonic tularemia challenges by both routes with Schu S4, the lungs of infected mice showed lesions characterized by necrosis and neutrophilic inflammation of the parenchyma and pleura. The lesions in the spleens were additionally characterized by multifocal and random necrosis. The BALB/c mice were extremely sensitive to challenge by the intranasal route with Schu S4 as the LD_50_ was determined to be 1–2 CFUs. In contrast, mice that were intranasally challenged with the CipR strain or the *kdsD*::*ltrB*_*L1*_ mutant were highly resistant (Table 6). However, when the intranasal challenge doses for the CipR mutant reached the range of ~10,000 CFUs, 3/10 mice did succumb by Day 16. Nevertheless, this is a much later time point than observed with mice challenged with wild-type Schu S4 which succumbed to infection by Day 5 when exposed to 300 CFU. In contrast, no mice challenged with the *kdsD*::*ltrB*_*L1*_ mutant succumbed to infection or showed any outward clinical signs, even at the highest challenge dose (82,000 CFU).

Thus, the *kdsD*::*ltrB*_*L1*_mutant does appear to be slightly more affected for virulence by pneumonic challenge models even though both the CipR and *kdsD* mutant strains are highly attenuated. This was also reflected in the pathology results for the aerosol challenged mice where a slight increase in lung necrosis and inflammation was noted in the lungs of the CipR mutant challenged mice as compared to the *kdsD*::*ltrB*_*L1*_ mutant challenged mice. In addition, the mice challenged with the *kdsD*::*ltrB*_*L1*_ mutant were estimated to have received over twice the dose as compared to the CipR mutant challenged mice. The exact reasoning for this slight difference in the level of attenuation between CipR and *kdsD*::*ltrB*_*L1*_ mutants remains to be determined and was unexpected as the CipR strain contains additional genetic changes. Potential reasons for this difference could be that the CipR mutant challenged mice lead to a hyperinduction of the immune response leading to increased damage since these bacteria remain longer in the lungs (Fig 7).

Other LPS (*waaY* and *waaL*) mutants in a Schu S4 background showed similar high levels of attenuation in BALB/c mice following intranasal challenge as determined by LD_50_ measurements (1.3x10^4^ and 3x10^3^, respectively) [66]. However, we noted several differences in the infection of BALB/c mice between the mutant strains. The Schu S4 *waaY* and *waaL* mutant strains examined by Rasmussen et al [66] both appeared to have induced a higher level of lung necrosis when mice were challenged with 10^6^ CFU of either mutant strain. In contrast, the *kdsD*::*ltrB*_*L1*_ mutant in our study showed less severe pneumonia early on following intranasal challenge compared to infection with the parent Schu S4 strain albeit, our challenge dose was with the *kdsD*::*ltrB*_*L1*_ mutant was several logs lower (6x10^3^ CFU). The *waaY* and *waaL* Schu S4 mutants were also able to disseminate from the lungs and replicate within the mouse organs examined [66]. In our study, the *kdsD*::*ltrB*_*L1*_ mutant was recovered poorly from the spleens following intranasal challenge, and CFUs recovered from the lungs did not increase over the time course examined.

Two possibilities might explain the differences between the studies. Firstly, the *waaY* and *waaL F*. *tularensis* mutants expressed a truncated LPS structure and retained a partial O-antigen structure [66]. In contrast, the *kdsD*::*ltrB*_*L1*_ mutant is unable to synthesize KDO; therefore expression of a complete core or O-antigen structure is greatly hindered (Fig 2). The *kdsD*::*ltrB*_*L1*_ mutant with a more dramatic LPS deficiency is likely more attenuated. Secondly, the doses used for the mouse challenges with the mutant strains in the two studies differed (10^6^ CFUs for *waaY* and *waaL* versus 10^3^ CFUs for *kdsD*::*ltrB*_*L1*_) therefore making a direct comparison in histopathology difficult.

Other LPS mutants of *F*. *tularensis* have demonstrated the potential to provide a protective response against parent strain challenges [66, 68, 97, 99–101]. Future vaccination studies with the Schu S4 *kdsD*::*ltrB*_*L1*_ mutant may be warranted to determine if some level of protection could be provided against challenge with fully virulent *F*. *tularensis* strain. Although, as detailed above, the dissemination and recovery of the *kdsD*::*ltrB*_*L1*_ mutant post-challenge was rather limited (Fig 7) as compared to the dissemination of other Schu S4 LPS mutant strains which allowed protection [66]. Therefore, efficacy of the *kdsD*::*ltrB*_*L1*_ mutant as a live vaccine could be limited.

However, a more promising avenue to explore is the potential of _D_- arabinose 5-phosphate isomerase (KdsD) to serve as a therapeutic target as previously proposed [102, 103]. This enzyme would be an attractive target for novel therapeutics against gram-negative bacteria as KDO is necessary for LPS synthesis and bacterial virulence. In addition, A5P would not be present within mammalian tissues to provide an exogenous source to infecting gram-negative bacteria. Furthermore, structural analyses studies have identified putative active sites for catalysis [104, 105], opening the possibility for the screening of small molecule inhibitors for drug design. Such novel targets and mechanisms of action are currently needed to combat antimicrobial resistance.

## Supporting information

S1 Fig Complementation of growth. *F*. *tularensis* (A) or *F*. *novicida* (B) strains were grown in Chamberlain’s defined medium (CDM) at 37^°^C. Growth was monitored by optical density. OD measurements were based upon quadruplicate samples and bars represent standard error of the mean. The *F*. *tularenesis kdsD*::*ltrB*_*L1*_ and *F*. *novicida kpsF*::T20 mutants were severely altered for growth in CDM. However, when a functional *kdsD* gene was supplied in trans on a plasmid to the mutants, growth was restored to the complemented strains.
(TIF)

S2 Fig Pathology of mice challenged intranasally with *F*. *tularensis* 1 day post-challenge. Mice were challenged intranasally with Schu S4 WT, CipR mutant, or *kdsD*::*ltrB*_*L1*_. The strains used for challenge and hematoxylin and eosin (HE) stained organ (lung, spleen, and liver) are as indicated.
(TIF)

S3 Fig Pathology of mice challenged intranasally with *F*. *tularensis* 3 days post-challenge. (TIF)

S4 Fig Pathology of mice challenged intranasally with *F*. *tularensis* 28 day post-challenge. Only CipR and *kdsD*::*ltrB*_*L1*_ mutant challenged mice survived to the end of the study.
(TIF)
