# Supplementary figures and images for: A spontaneous mutation in kdsD, a biosynthesis gene for 3 Deoxy-D-manno-Octulosonic Acid, occurred in a ciprofloxacin resistant strain of Francisella tularensis and caused a high level of attenuation in murine models of tularemia
Source: PLoS One. 2017 Mar 22;12(3):e0174106. doi: 10.1371/journal.pone.0174106 (PMC5362203; doi:10.1371/journal.pone.0174106)

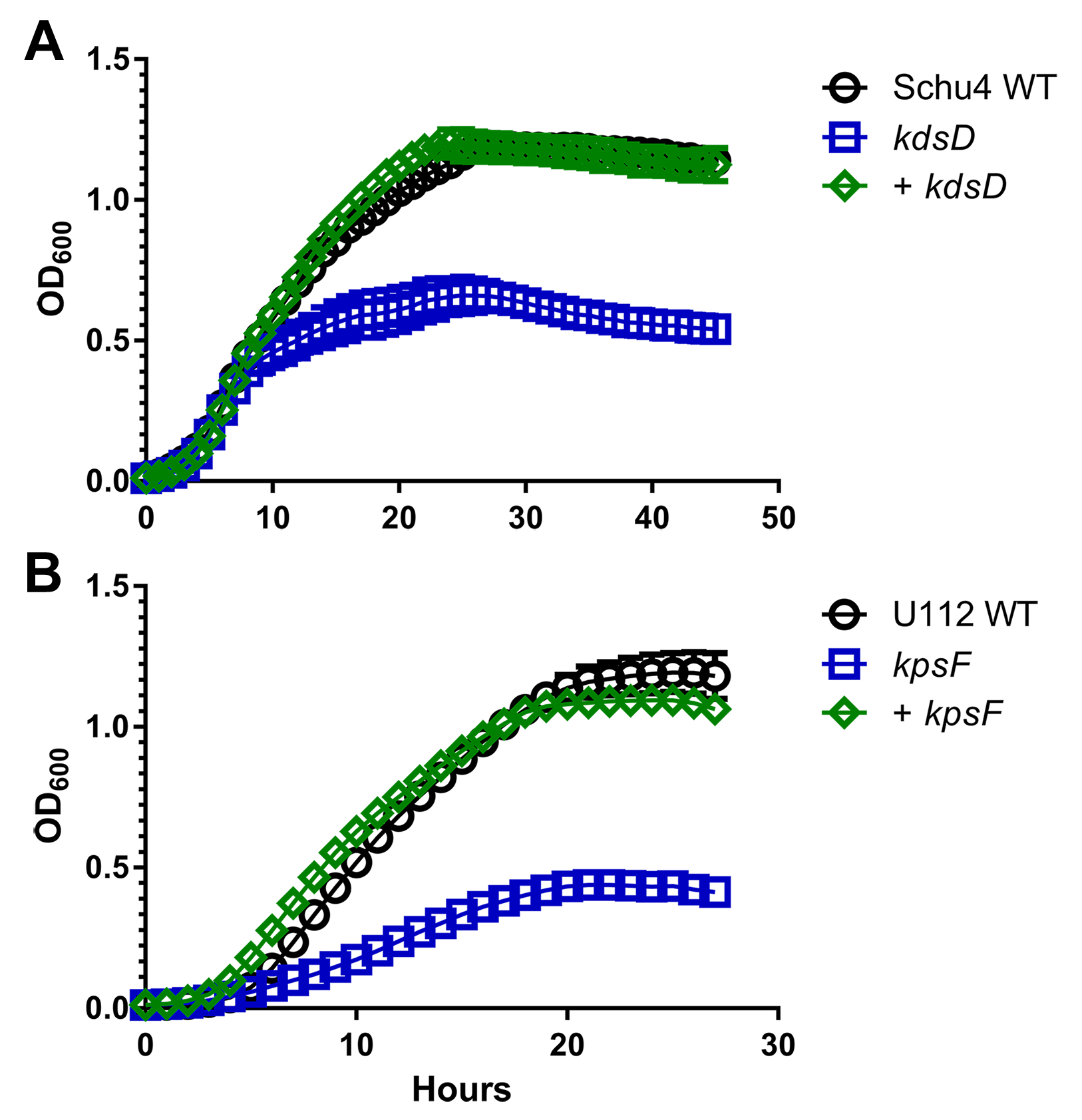

Supplement: S1 Fig — F. tularensis (A) or F. novicida (B) strains were grown in Chamberlain’s defined medium (CDM) at 37°C. Growth was monitored by optical density. OD measurements were based upon quadruplicate samples and bars represent standard error of the mean. The F. tularenesis kdsD::ltrBL1 and F. novicida kpsF::T20 mutants were severely altered for growth in CDM. However, when a functional kdsD gene was supplied in trans on a plasmid to the mutants, growth was restored to the complemented strains. (TIF) [file pone.0174106.s001.tif]

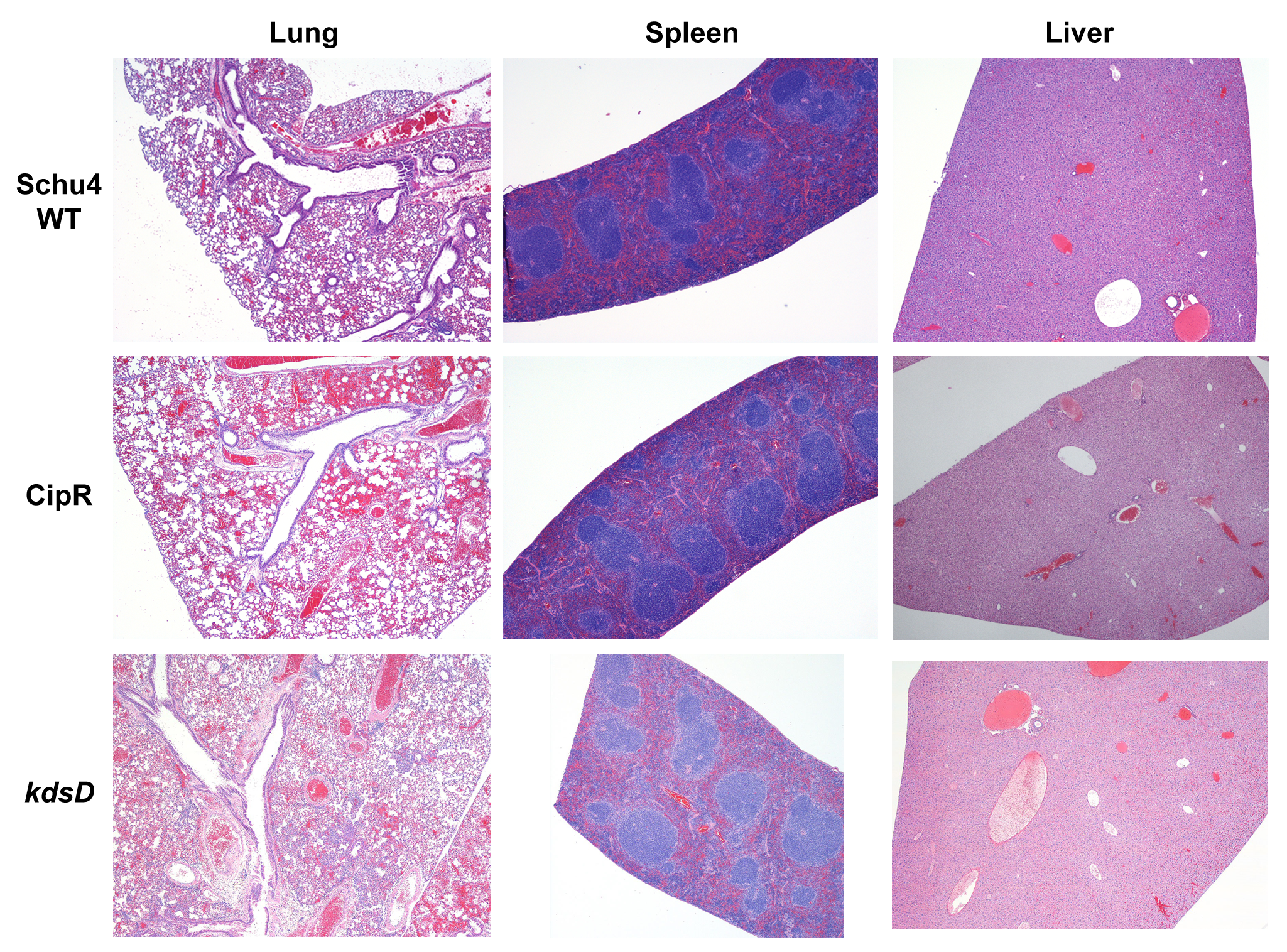

Supplement: S2 Fig — Mice were challenged intranasally with Schu S4 WT, CipR mutant, or kdsD::ltrBL1. The strains used for challenge and hematoxylin and eosin (HE) stained organ (lung, spleen, and liver) are as indicated. (TIF) [file pone.0174106.s002.tif]

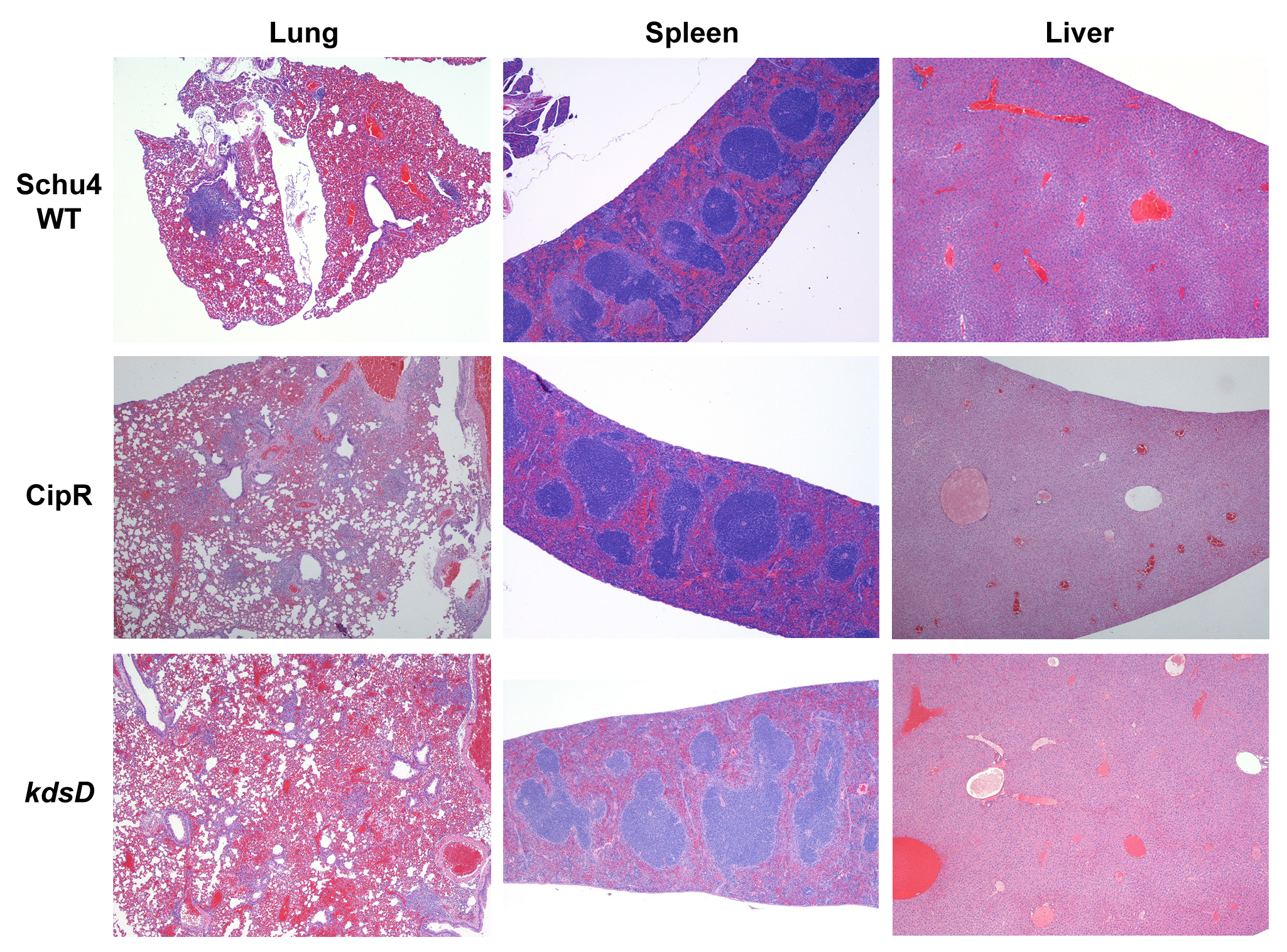

Supplement: S3 Fig — (TIF) [file pone.0174106.s003.tif]

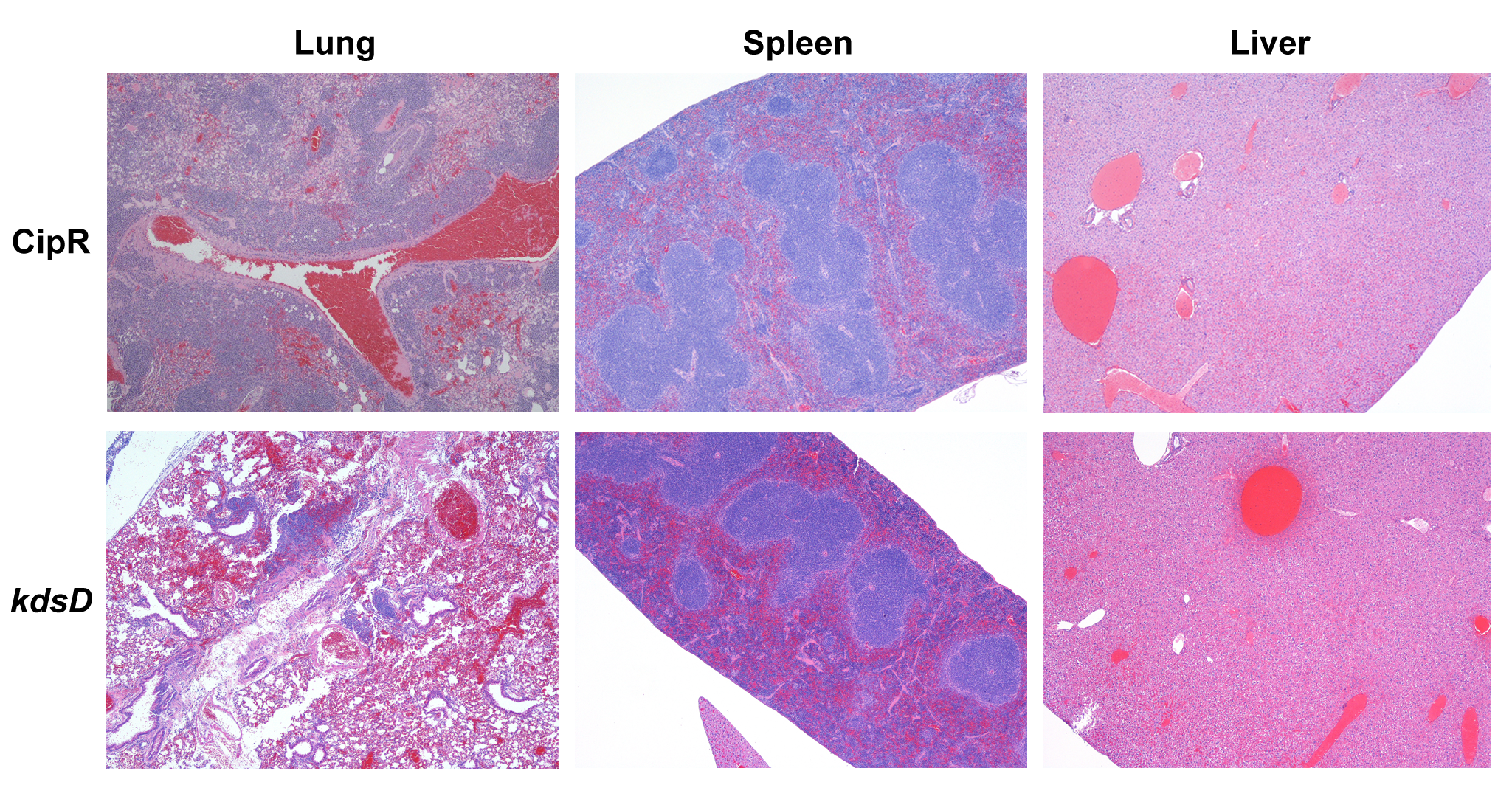

Supplement: S4 Fig — Only CipR and kdsD::ltrBL1 mutant challenged mice survived to the end of the study. (TIF) [file pone.0174106.s004.tif]
